# B-lymphoid tyrosine kinase-mediated FAM83A phosphorylation elevates pancreatic tumorigenesis through interacting with β-catenin

**DOI:** 10.1038/s41392-022-01268-5

**Published:** 2023-02-17

**Authors:** Cefan Zhou, Xiaoting Zhu, Nanxi Liu, Xueying Dong, Xuewen Zhang, Huili Huang, Yu Tang, Shicheng Liu, Mengyu Hu, Ming Wang, Xiaoling Deng, Shi Li, Rui Zhang, Yuan Huang, Hao Lyu, Shuai Xiao, Sang Luo, Declan William Ali, Marek Michalak, Xing-Zhen Chen, Zhentian Wang, Jingfeng Tang

**Affiliations:** 1 National “111” Center for Cellular Regulation and Molecular Pharmaceutics, Key Laboratory of Fermentation Engineering (Ministry of Education), Hubei University of Technology, Wuhan, 430068 China; 2 Membrane Protein Disease Research Group, Department of Physiology, Faculty of Medicine and Dentistry, University of Alberta, Edmonton, AB T6G2R3 Canada; 3 Cooperative Innovation Center of Industrial Fermentation (Ministry of Education & Hubei Province), Hubei Key Laboratory of Industrial Microbiology, Hubei University of Technology, Wuhan, China; 4 Department of Systems Biology for Medicine, School of Basic Medical Sciences, Fudan University, and Shanghai Fifth People’s Hospital, Fudan University, Shanghai, 200433 China; 5 Department of Clinical Laboratory, Renmin Hospital of Wuhan University, Wuhan, 430060 China; 6 Ningxia Key Laboratory of Stem Cell and Regenerative Medicine, General Hospital of Ningxia Medical University, Ningxia, 750001 China; 7 Department of Biological Sciences, University of Alberta, Edmonton, AB T6G2R3 Canada; 8 Department of Biochemistry, University of Alberta, Edmonton, AB T6G2R3 Canada

**Keywords:** Molecular medicine, Oncogenes, Molecular biology

## Abstract

Abnormal activation of Wnt/β-catenin-mediated transcription is closely associated with the malignancy of pancreatic cancer. Family with sequence similarity 83 member A (FAM83A) was shown recently to have oncogenic effects in a variety of cancer types, but the biological roles and molecular mechanisms of FAM83A in pancreatic cancer need further investigation. Here, we newly discovered that FAM83A binds directly to β-catenin and inhibits the assembly of the cytoplasmic destruction complex thus inhibiting the subsequent phosphorylation and degradation. FAM83A is mainly phosphorylated by the SRC non-receptor kinase family member BLK (B-lymphoid tyrosine kinase) at tyrosine 138 residue within the DUF1669 domain that mediates the FAM83A-β-catenin interaction. Moreover, FAM83A tyrosine 138 phosphorylation enhances oncogenic Wnt/β-catenin-mediated transcription through promoting β-catenin-TCF4 interaction and showed an elevated nucleus translocation, which inhibits the recruitment of histone deacetylases by TCF4. We also showed that FAM83A is a direct downstream target of Wnt/β-catenin signaling and correlates with the levels of Wnt target genes in human clinical pancreatic cancer tissues. Notably, the inhibitory peptides that target the FAM83A-β-catenin interaction significantly suppressed pancreatic cancer growth and metastasis in vitro and in vivo. Our results revealed that blocking the FAM83A cascade signaling defines a therapeutic target in human pancreatic cancer.

## Introduction

Pancreatic ductal adenocarcinoma (PDAC) is a lethal disease with a 5-year survival rate of 5%.^1^ Approximately 80% of pancreatic cancer patients present with locally advanced or metastatic disease at the time of diagnosis and thus are not eligible for surgery. Although diagnostic and surgical treatment methods have been developed to aid in lengthening survival and providing symptom relief, few approaches provide a curative effect.^2^ Moreover, both intrinsic and acquired chemo-resistant behaviors are major factors that significantly decrease the clinical efficacy of chemotherapy for pancreatic cancer.^3^ Understanding the biology of pancreatic cancer and identifying its putative therapeutic targets in the dysregulated oncogenic pathways are urgently needed for PDAC patients.

Mutations that result in the constitutively activated Wnt/β-catenin signal pathways are thought to be related to pancreatic cancer progression.^4^ β-catenin is the central effector that mediates the transcription activity of numerous oncogenic target genes, such as C-myc, CyclinD1, and AXIN2. Cytoplasmic β-catenin is constitutively phosphorylated for degradation by the destruction complex, which consists of two multidomain scaffolding proteins AXIN1, adenomatous polyposis coli (APC), glycogen synthase kinase 3β (GSK3β) and casein kinase Iα (CK1α) without Wnt stimulation. Phosphorylated β-catenin is then recognized by the E3 ubiquitin ligase β-transducin repeat-containing protein (β-TrCP) and is thus ubiquitinated and degraded by proteasome.^5^ Moreover, in the absence of Wnt/β-catenin signaling, Wnt target genes are silenced by co-repressors of Groucho/TLE1 and histone deacetylase (HDAC).^6^ Upon Wnt stimulation, the destruction complex is dissociated, and the stabilized β-catenin subsequently enters the nucleus and activates the transcription of downstream target genes via the T-cell factor (TCF)/lymphoid enhancer-binding factor (LEF) of transcription factors.^7^

Family with sequence similarity 83 member A (FAM83A) was originally identified as BJ-TSA-9, which is highly expressed in lung cancer, without known function(s).^8^ FAM83A is a 434-amino-acid protein and contains DUF1669, serine-rich domains, and proline-rich domains (PRDs).^9^ A previous study has reported that FAM83A is overexpressed in multiple human tumors, such as lung, cervix, liver, and pancreatic cancer,^10–13^ suggesting a tumorigenesis role of FAM83A during cancer development and progression. The UniProt database describes that FAM83A independently functions in activating the PI3K/AKT signaling cascade pathway.^11^ Moreover, FAM83A has been identified as a candidate cancer-associated gene capable of conferring resistance on EGFR-tyrosine kinase inhibitors (TKIs) through interacting with and causing phosphorylation of c-RAF and PI3K p85, upstream of MAPK and downstream of EGFR.^9^ Notably, recent studies have indicated that FAM83A promotes the proliferative and invasive abilities of cancer cells via epithelial-mesenchymal transition and the Wnt signaling pathway.^14,15^ However, the detailed molecular mechanisms remain to be investigated.

In the present study, we figured out the roles of FAM83A and its posttranslational modification in states in canonical Wnt/β-catenin signaling regulation. Mechanically, we demonstrated that FAM83A interacts with β-catenin and acts as a negative component of the cytoplasmic β-catenin destruction complex that inhibits its assembly. We also identified that the FAM83A protein is phosphorylated by the SRC kinase subfamily member BLK at the tyrosine 138 residue, where the phosphorylation level markedly elevates the oncogenic potential of pancreatic cancer cells. Moreover, the peptides that target the FAM83A and β-catenin interaction significantly suppress the activity of canonical Wnt/β-catenin signaling and restrain the proliferation and metastasis of pancreatic cancer cells.

## Results

### FAM83A is identified as a new component of the cytoplasmic β-catenin destruction complex

**Fig. 1:**
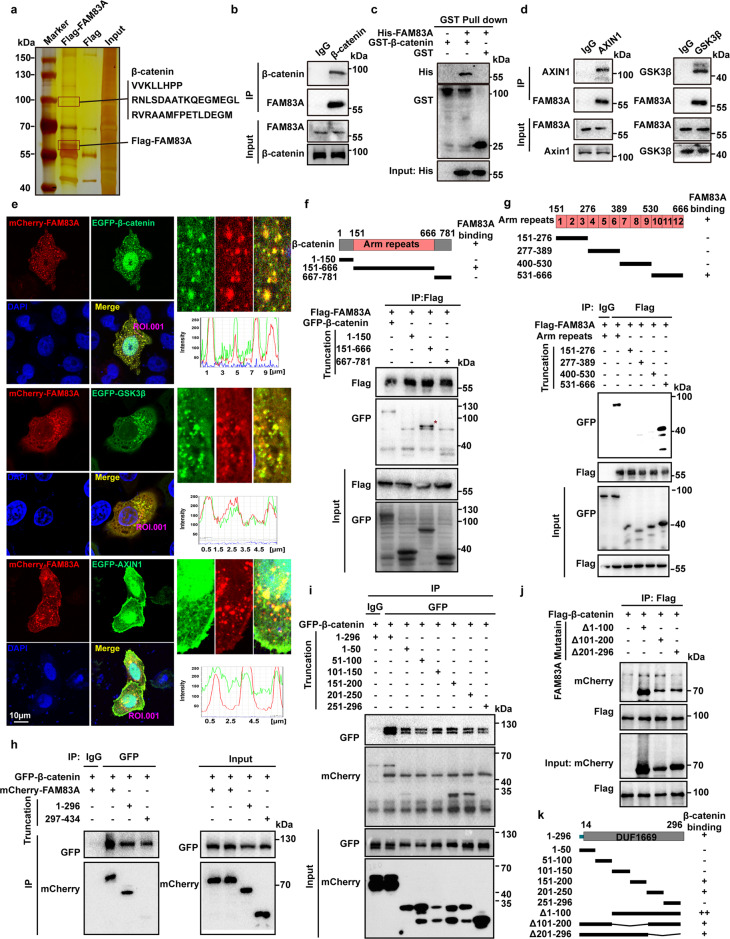
FAM83A is identified as a new component of the cytoplasmic β-catenin destruction complex. **a** Silver staining of immunoprecipitate using Flag antibody from HEK293T cell lysates transfected with Flag-tagged FAM83A. **b** The interaction between endogenous FAM83A and β-catenin in pancreatic cancer PANC-1 cells. Protein interactions were analyzed by western blotting. **c** Externally purified GST-tagged full-length β-catenin was incubated with externally purified His-tagged FAM83A, the mixture was pulled down by GST beads and then the precipitates were subjected to western blotting. **d** The interaction between endogenous FAM83A and the components of β-catenin destructive complex AXIN1 and GSK3β in PANC-1 cells. Protein interactions were analyzed by western blotting. **e** The co-localization of mCherry-tagged FAM83A and EGFP-tagged β-catenin, GSK3β, and AXIN1 in HeLa cells were analyzed with confocal microscopy. **f**, **g** HEK293T cells were transfected with the β-catenin truncation mutations indicated for 48 h and then harvested for IP and western blotting analysis. The schematic diagram of β-catenin truncation generation and FAM83A-β-catenin mutants interacting domain were shown. **h**–**k** HEK293T cells were transfected with the FAM83A truncation or deletion mutations indicated for 48 h and then harvested for IP and western blotting analysis. The schematic diagram of FAM83A truncation and deletion generation and β-catenin-FAM83A mutants interacting domain were shown. Scale bars: 10 µm

To investigate the mechanisms of FAM83A in pancreatic cancer progression, a Flag-tag affinity procedure was performed to purify a FAM83A-containing complex in HEK293T cells, which was then subjected to LS-MS/MS analysis. The results showed that three peptide sequences of β-catenin were found in the complex, which suggests that β-catenin is a potential binding partner of FAM83A (Fig. 1a). The interaction between FAM83A and β-catenin was confirmed using immunoprecipitation analysis in pancreatic cancer PANC-1 cells and 293T cells transfected with Flag-tagged FAM83A and HA-tagged β-catenin (Fig. 1b, Supplementary Fig. S1a). The physical interactions between β-catenin and FAM83A were further analyzed in vitro using recombinant GST-β-catenin and His-FAM83A. The result indicated that purified His-FAM83A bound directly to GST-β-catenin (Fig. 1c). Based on the finding that FAM83A interacts with β-catenin, we asked whether FAM83A is a member of the β-catenin destruction complex. Indeed, FAM83A formed a complex with GSK3β in an exogenous coimmunoprecipitation (co-IP) assay (Supplementary Fig. S1b). In endogenous co-IP assay and confocal fluorescence microscope image, the coexistence of GSK3β and FAM83A, AXIN1, and FAM83A within a complex were observed (Fig. 1d, e). The β-catenin protein consists of an NH2-terminal domain, a central armadillo (Arm) repeats domain (residues 151–666) composed of 12 Arm repeats, and a COOH-terminal domain (residues 667–782) (Fig. 1f, g, up). Using a series of GFP-tagged β-catenin deletion mutants, we found that Arm repeats 10–12 of β-catenin interacted with FAM83A (Fig. 1f, g, down). FAM83A consists of an NH2-terminal domain, a conserved domain of the unknown function (DUF1669) (residues 14–296), and a conserved serine-rich and proline-rich domain (residues 320–394), and a COOH-terminal domain (Supplementary Fig. S1c, up). We found that the DUF1669 domain of FAM83A interacted with β-catenin (Fig. 1h). Additionally, we also found that the DUF1669 domain-mediated the interactions between FAM83A and AXIN1, GSK3β (Supplementary Fig. S1c, d) and FAM83A binds the kinase domain of GSK3β (Supplementary Fig. S1e). We further document the minimum domain of FAM83A that binds β-catenin using a series of mCherry-tagged FAM83A mutants containing approximately 50 amino-acid (aa) residues. The results revealed that aa 151–250 within the DUF1669 domain-mediated the interaction between FAM83A and β-catenin (Fig. 1i–k). These results indicate that FAM83A is a new component of the cytoplasmic β-catenin destruction complex.

### FAM83A promotes canonical β-catenin transcriptional activity in human pancreatic cancer

**Fig. 2:**
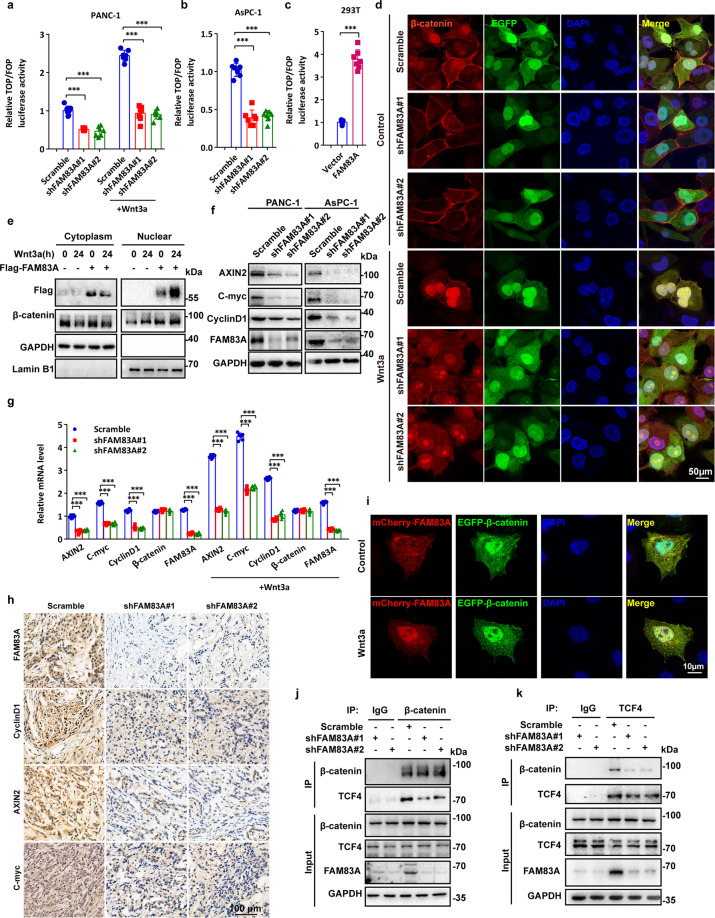
FAM83A promotes canonical β-catenin transcriptional activity in human pancreatic cancer. **a**–**c** Wnt reporter luciferase activity (TOP Flash) in PANC-1, AsPC-1, and HEK293T cells with or without FAM83A knockdown or overexpression and Wnt3a treatment (100 ng/mL for 4 hours) (*n* = 7). **d** The localization of β-catenin with or without FAM83A knockdown (cells expressing FAM83A shRNA co-expressed GFP) and Wnt3a treatment (100 ng/mL for 4 hours) were analyzed with confocal microscopy in PANC-1 cells. **e** The protein level of cytoplasmic and nuclear β-catenin in HEK293T cells with or without Wnt3a treatment (100 ng/mL) were analyzed with western blotting (*n* = 3). **f** (*n* = 3). **g** mRNA level of Wnt target genes AXIN2, C-myc, and CyclinD1 in PANC-1 cell lysates with or without FAM83A knockdown and Wnt3a treatment (100 ng/mL) (*n* = 6). **h** Representative immunohistochemical images of FAM83A and Wnt target genes AXIN2, C-myc, and CyclinD1 expression in excised xenograft tumor tissues with or without FAM83A knockdown. **i** The localization of mCherry-tagged FAM83A and GFP-tagged β-catenin with or without Wnt3a treatment (100 ng/mL) in PANC-1 cells was analyzed with confocal microscopy. **j**, **k** FAM83A knockdown decreased the interaction between endogenous TCF4 and β-catenin in PANC-1 cell lysates. Protein interactions were analyzed by western blotting (*n* = 3). Scale bars: 10 µm. *P < 0.05; **P < 0.01; ***P < 0.001. Data were presented as mean ± SD

The previously described findings prompted us to investigate whether FAM83A affects Wnt/β-catenin signaling. We found that FAM83A knockdown significantly decreased the basal and Wnt3a-induced transactivating activity of β-catenin in PANC-1 and AsPC-1 cells, as determined by a TCF/β-catenin regulated reporter (TOPflash) and a mutant (FOPflash) reporter assay (Fig. 2a, b). Conversely, FAM83A overexpression increased the TOPflash activity in HEK293T cells (Fig. 2c). Similar results were found in another EGFP and mCherry tandem labeled TCF/LEF transcription element system (7TGC) in PANC-1 cells (Supplementary Fig. S2a). Moreover, FAM83A depletion reduced β-catenin nuclear localization in basal and Wnt3a treated conditions (Fig. 2d, Supplementary Fig. S2b). Overexpression of FAM83A decreased the amount of cytoplasmic, and increased nuclear β-catenin with or without Wnt3a treatment (Fig. 2e). Both the protein and mRNA expression of genes regulated by Wnt/β-catenin signalings, such as AXIN2, C-myc, and CyclinD1, were significantly decreased while FAM83A was depleted (Fig. 2f, g). However, FAM83A depletion had no effects on β-catenin mRNA level (Fig. 2g). We also confirmed the levels of AXIN2, C-myc, and CyclinD1 in excised xenograft tumors originating from pancreatic cancer cells harboring FAM83A knockdown were markedly downregulated (Fig. 2h). Additionally, to our surprise, the results from Fig. 2e, i also showed that FAM83A shifted from the cell cytoplasm to nucleus upon Wnt3a treatment. Because the interaction between FAM83A and β-catenin was mediated by the region of β-catenin (Arm10–12), which contains the amino acids critical to its binding with TCF4 (Arm10),^16^ we examined whether FAM83A affects the ability of TCF4 to recruit β-catenin. The results revealed that FAM83A depletion decreased the interaction between β-catenin and TCF4, whereas FAM83A overexpression enhanced β-catenin-TCF4 complex formation (Fig. 2j, k, Supplementary Fig. S2c). Collectively, these data indicate that FAM83A is a positive regulator of the Wnt/β-catenin signaling pathway.

### FAM83A inhibits the destruction complex formation and stabilizes β-catenin

**Fig. 3:**
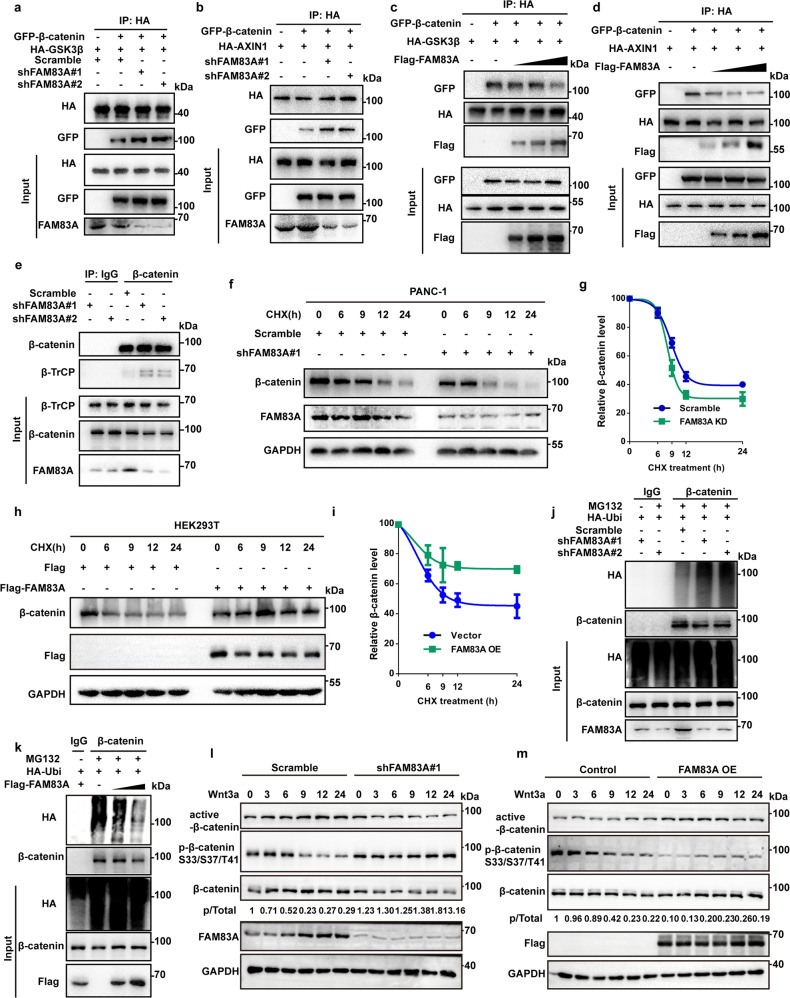
FAM83A inhibits the destruction complex formation and stabilizes β-catenin. **a**, **b** FAM83A knockdown using two specific small interfering RNAs (shFAM83A#1 and shFAM83A#2) enhanced the interaction between exogenous HA-tagged GSK3β, AXIN1 and GFP-tagged β-catenin in PANC-1 cell lysates. Protein interactions were analyzed by western blotting (*n* = 3). **c**, **d** The exogenous Flag-FAM83A dose-dependently decreased β-catenin-GSK3β and β-catenin-AXIN1 interactions in PANC-1 cell lysates. Protein interactions were analyzed by western blotting (*n* = 3). **e** FAM83A knockdown enhanced the interaction between endogenous β-TrCP and β-catenin in PANC-1 cell lysates. Protein interactions were analyzed by western blotting (*n* = 3). **f**, **g** FAM83A knockdown promoted the degradation of β-catenin protein upon cycloheximide (CHX, 20 μM) treatment for indicated times in PANC-1 cell lysates. Representative western blotting images and quantification data were shown (*n* = 3). **h**, **i** FAM83A overexpression inhibited the degradation of β-catenin protein upon cycloheximide (CHX, 20 μM) treatment for indicated times in PANC-1 cell lysates. Representative western blotting images and quantification data were shown (*n* = 3). **j** FAM83A knockdown increased the level of β-catenin ubiquitination upon MG132 (10 μM) treatment in PANC-1 cell lysates. Representative western blotting images were shown (*n* = 3). **k** FAM83A overexpression dose-dependently decreased the level of β-catenin ubiquitination upon MG132 (10 μM) treatment in PANC-1 cell lysates. Representative western blotting images were shown (*n* = 3). **l**, **m** Representative western blotting images of active-β-catenin, p-β-catenin S33/S37/T41 were shown after FAM83A knockdown or FAM83A overexpression upon Wnt3a (100 ng/mL) treatment for indicated times (*n* = 3). *P < 0.05; **P < 0.01; ***P < 0.001. Data were presented as mean ± SD

To investigate the functions of FAM83A in the β-catenin destruction complex and β-catenin stability, we asked whether FAM83A influences the stability of the destruction complex. To this end, the interaction between GSK3β, AXIN1, and β-catenin was evaluated. The results showed that FAM83A depletion increased the binding activity of either GSK3β or AXIN1 to β-catenin, whereas FAM83A overexpression reduced the association between GSK3β or AXIN1 and β-catenin in a dose-dependent manner in HEK293T, PANC-1 and SW1990 cells (Fig. 3a–d, Supplementary Fig. S2d). Moreover, we also found that FAM83A knockdown increased the recruitment of β-catenin ubiquitin E3 ligase β-TrCP to β-catenin (Fig. 3e). To examine the effects of FAM83A on the protein level of total β-catenin, PANC-1, and HEK293T cells were incubated with cycloheximide, which could prevent the synthesis of new β-catenin. The results revealed that FAM83A knockdown significantly accelerated the degradation of β-catenin, resulting in a marked decrease in the amount of β-catenin (Fig. 3f, g). In contrast, FAM83A expression restrained the decrease of β-catenin protein levels in HEK293T cells (Fig. 3h, i). Moreover, the influence of FAM83A on the ubiquitination and phosphorylation status of β-catenin was also assessed. The results showed that FAM83A depletion increased β-catenin ubiquitination as well as phosphorylation on Ser33/Ser37/Thr41. Consistently, the ubiquitination and phosphorylation levels of β-catenin decreased in FAM83A overexpression conditionally (Fig. 3j–m). Additionally, FAM83A depletion decreased, whereas FAM83A overexpression increased active β-catenin levels (Fig. 3l, m, Supplementary Fig. S2e). Taken together, these data indicate that FAM83A inhibits the destruction of complex formation and stabilizes β-catenin in the cell cytoplasm.

### FAM83A is phosphorylated by BLK kinase at Y138

**Fig. 4:**
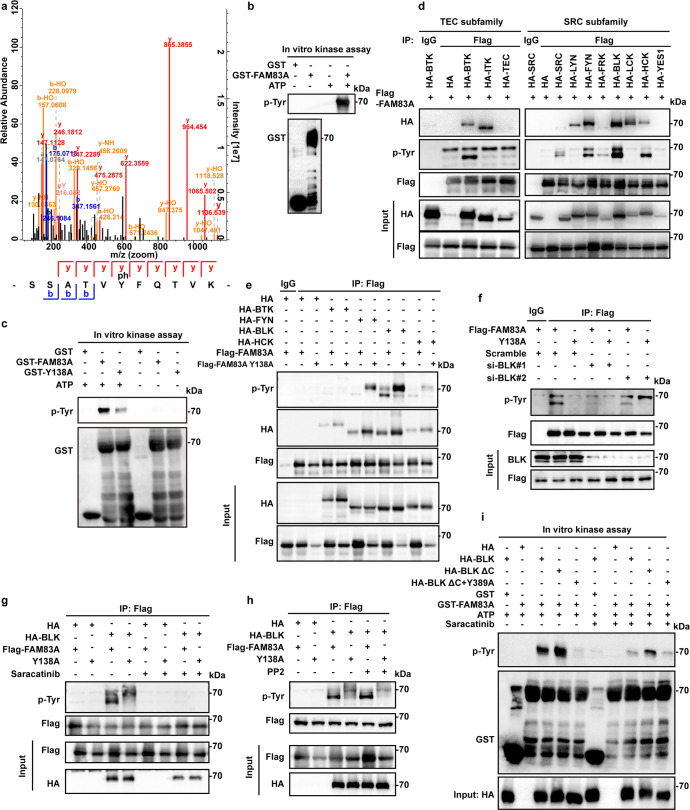
FAM83A is phosphorylated by BLK kinase at Y138. **a** The mass spectrum showed that FAM83A extracted from HEK293T cell lysates was phosphorylated at tyrosine 138 residue. **b** In vitro purified GST-FAM83A were incubated with HEK293T cell lysates with or without ATP addition, and the reaction products were subjected to western blotting. **c** In vitro purified GST-tagged FAM83A and GST-tagged FAM83A Y138A mutant were incubated with HEK293T cell lysates with or without ATP addition, and the reaction products were subjected to western blotting (*n* = 3). **d** The in vivo interaction between Flag-tagged FAM83A and the components of non-receptor TEC kinase subfamily or SRC kinase subfamily were analyzed in HEK293T cells by western blotting (*n* = 3). **e** Level of total tyrosine phosphorylation of Flag-tagged FAM83A and its Y138A mutant in HEK293T cells after HA-tagged BTK, FYN, BLK, and HCK transfection. Cell lysates were used for IP and western blotting with the indicated antibodies (*n* = 3). **f** Level of total tyrosine phosphorylation of Flag-tagged FAM83A and its Y138A mutant in HEK293T cells after BLK kinase knockdown using two specific small interfering RNAs (siBLK#1 and siBLK#2). Cell lysates were used for IP and western blotting with the indicated antibodies (*n* = 3). **g**, **h** Level of total tyrosine phosphorylation of Flag-tagged FAM83A and its Y138A mutant in HEK293T cells with or without BLK kinase overexpression and saracatinib (BLK inhibitor, 10 μM)/PP2 (LCK or FYN inhibitor, 10 μM) treatment. Cell lysates were used for IP and western blotting with the indicated antibodies (*n* = 3). **i** The BLK kinases was obtained from the immunoprecipitate of HEK293T cell lysates that transfected with either HA-tagged BLK wild-type, BLK kinase-dead mutant (Y389A) or BLK constitutive activated mutant with the deletion of the C-terminal auto-inhibitory domain (BLK ΔC) using HA tag antibody. Then the BLK kinases were incubated with the in vitro purified GST-tagged FAM83A protein at 30 °C for 30 min with or without BLK inhibitor saracatinib, and then the reaction products were subjected to western blotting assay (*n* = 3)

From our mass spectrometry data shown in Fig. 1a, we identified that FAM83A is phosphorylated at tyrosine (Tyr, Y) 138 residue (Fig. 4a). In vitro kinase assays using the purified GST-tagged FAM83A and PANC-1 cell lysates revealed that FAM83A was indeed phosphorylated at tyrosine residues using an anti-phosphoTyr antibody (Fig. 4b). Moreover, we also replaced Y138 with alanine (A) to generate a non-phosphorylatable mutant and found that the purified GST-FAM83A Y138A mutant significantly reduced tyrosine phosphorylation in the presence of adenosine triphosphate (ATP) (Fig. 4c). Given that some kinds of non-receptor kinase, such as FYN, BLK, and SRC, were identified in the FAM83A immunoprecipitate from the LS-MS/MS data, we next determined the kinases that are responsible for the phosphorylation of the FAM83A protein. To this end, we screened two major non-receptor tyrosine kinase family members, the SRC and TEC kinase subfamily, for their role in FAM83A phosphorylation. The immunoprecipitation analysis showed that the TEC subfamily member BTK and the SRC subfamily members SRC, FYN, BLK, and HCK were responsible for the FAM83A protein tyrosine phosphorylation, whereas the FAM83A Y138A mutant showed rare phosphotyrosine signals. Specifically, BLK kinase showed the most robust effect on FAM83A phosphorylation (Fig. 4d, e). To further verify the role of BLK in FAM83A phosphorylation in vivo, we knocked down BLK expression using two short interfering RNAs (siRNAs). The results revealed that BLK knockdown diminished FAM83A tyrosine phosphorylation (Fig. 4f). Moreover, BLK-mediated FAM83A tyrosine phosphorylation could be reduced by the BLK kinase inhibitor saracatinib, not LYN kinase inhibitor PP2 (Fig. 4g, h). Furthermore, the BLK-activated form, BLK ΔC, which lacks a portion of the C-terminal BLK region that encompasses the inhibitory autophosphorylation Y501 residue and the BLK kinase-dead mutant Y389A, which cannot bind ATP was constructed. The transient transfection of BLK ΔC markedly enhanced the phosphorylated level of FAM83A compared with the wild-type BLK, whereas the kinase-dead form, BLK Y398A, showed decreased FAM83A tyrosine phosphorylation levels without the treatment of BLK inhibitor saracatinib (Supplementary Fig. S3A). Additionally, similar results were found in the in vitro incubation assay of purified GST-FAM83A protein with the immunoprecipitated BLK ΔC and BLK Y398A mutants (Fig. 4i). These data indicate that FAM83A is mainly phosphorylated by BLK at Y138.

### Phosphorylated FAM83A inhibits β-catenin destruction complex assembly and HDAC recruitment by TCF4

**Fig. 5:**
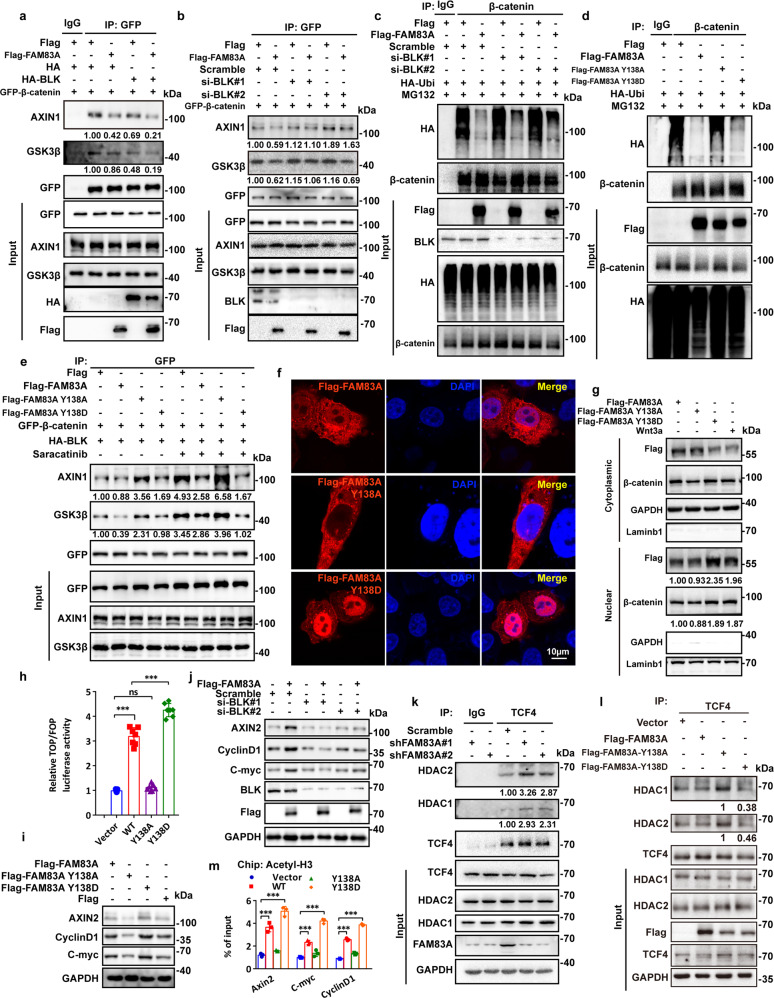
Phosphorylated FAM83A promotes β-catenin destruction complex assembly and inhibits HDAC recruitment by TCF4. **a**, **b** The interaction between GFP-tagged β-catenin and endogenous GSK3β and AXIN1 after BLK kinase overexpression or knockdown in PANC-1 cells. Protein interactions were analyzed by western blotting (*n* = 3). **c** The level of β-catenin ubiquitination with or without Flag-tagged FAM83A overexpression or BLK kinase knockdown after MG132 treatment in HEK293T cells. Cell lysates were used for IP and western blotting with the indicated antibodies (*n* = 3). **d** The level of β-catenin ubiquitination with or without Flag-tagged FAM83A, Y138A, or Y138D mutants overexpression after MG132 treatment in HEK293T cells. Cell lysates were used for IP and western blotting with the indicated antibodies (*n* = 3). **e** The interaction between GFP-tagged β-catenin and endogenous GSK3β and AXIN1 after BLK kinase overexpression with or without Flag-tagged FAM83A, Y138A, or Y138D mutants transfection or saracatinib treatment in HEK293T cells. Cell lysates were used for IP and western blotting with the indicated antibodies (*n* = 3). **f** The localization of Flag-tagged FAM83A and its Y138A, Y138D mutants in HEK293T cells were analyzed with confocal microscopy. **g** The protein level of cytoplasmic and nuclear β-catenin in HEK293T cells with or without Flag-tagged FAM83A, Y138A, or Y138D mutants transfection and Wnt3a treatment. Cell lysates were used for western blotting with the indicated antibodies (*n* = 3). **h**, **i** Wnt reporter luciferase activity (TOP Flash, *n* = 7) and the level of Wnt target genes AXIN2, C-myc, and CyclinD1 in HEK293T cells with or without Flag-tagged FAM83A, Y138A, or Y138D mutants transfection (*n* = 3). **j** Western blotting analysis of Wnt target genes AXIN2, C-myc, and CyclinD1 in PANC-1 cell lysates with or without FAM83A transfection and BLK kinase knockdown (*n* = 3). **k**, **l** The interaction between HDAC1, HDAC2 and TCF4 after FAM83A knockdown in PANC-1 cells or Flag-tagged FAM83A, Y138A or Y138D mutants transfection in HEK293T cells (*n* = 3). Cell lysates were used for IP and western blotting with the indicated antibodies. **m** The level of Wnt target genes AXIN2, C-myc and CyclinD1 in the PANC-1 cells stably expressed Flag-tagged FAM83A, Y138A or Y138D mutants in chromatin immunoprecipitation using histone H3 acetylation antibodies (*n* = 3). *P < 0.05; **P < 0.01; ***P < 0.001. Data were presented as mean ± SD

To investigate the roles of FAM83A phosphorylation in Wnt/β-catenin signaling, we first assessed whether FAM83A phosphorylation by BLK affected β-catenin destruction complex assembly. It is shown that BLK overexpression decreased the binding ability of GSK3β and AXIN1 to β-catenin, whereas FAM83A overexpression amplifies its effects. Moreover, BLK knockdown showed opposite effects (Fig. 5a, b, Supplementary Fig. S4a). We also found that BLK knockdown increased the level of β-catenin ubiquitination upon FAM83A overexpression (Fig. 5c). Consistently, the FAM83A Y138A mutant significantly increased the levels of β-catenin ubiquitination and the amount of AXIN1 or GSK3β in the β-catenin immunoprecipitate, whereas the Y138D mutant enhanced β-catenin protein stability and restrained destruction complex assembly (Fig. 5d, e, Supplementary Fig. S4b). Surprisingly, in the confocal images, we found wild-type FAM83A was co-localized in both the cytoplasm and nucleus, and Wnt3a increased the nuclear translocation of FAM83A (Fig. 2i, Fig. 5f, top), and the FAM83A Y138A and Y138D mutants were distributed in cytoplasm and nucleus, respectively (Fig. 5f). Another nucleo-plasmic separation experiment also confirmed this observation and showed that FAM83A contributed to β-catenin nuclear translocation (Fig. 5g).

Given that FAM83A promotes β-catenin-mediated transcriptional activity and nuclear localization, we further investigated whether FAM83A binds with TCF4 and whether its phosphorylation status affects TCF4-mediated transcription. The immunoprecipitation assay showed that FAM83A accurately interacted with TCF4 through its DUF1899 domain and the FAM83A Y138D mutant co-localized with TCF4 (Supplementary Fig. S4c, d). The FAM83A Y138A mutant significantly decreased, and Y138D increased the luciferase activity of TOPflash and the expression of the Wnt target genes, respectively (Fig. 5h, i). Moreover, we also confirmed that BLK knockdown showed inhibitory effects on the levels of AXIN2, CyclinD1, and C-myc (Fig. 5j). Previous studies have revealed that both TCF and β-catenin interact with HDACs, and histone acetylation was essential for TCF4/β-catenin-mediated transcription,^17^ we assessed whether FAM83A affected the recruitment of HDAC to the TCF4/β-catenin complex. The co-IP assays revealed that FAM83A depletion significantly increased the binding of endogenous HDAC1/2 with β-catenin or TCF4 (Fig. 5k, Supplementary Fig. S4e). In contrast, FAM83A overexpression decreased the amount of HDAC1/2 in the β-catenin immunoprecipitate in a dose-dependent manner (Supplementary Fig. S4f). Furthermore, the FAM83A Y138A mutant showed enhanced effects on the interaction between HDAC1/2 and TCF4, whereas the Y138D mutant showed the opposite effect (Fig. 5l). Additionally, a ChIP assay with an anti-total acetylated histone H3 antibody demonstrated that FAM83A phosphorylation enhanced histone H3 acetylation in the promoter region of the Wnt target genes AXIN2, CyclinD1, and C-myc compared with wild-type FAM83A (Fig. 5m). These data indicate that the phosphorylated FAM83A inhibits β-catenin destruction complex assembly and HDAC recruitment by TCF4/β-catenin complex.

### FAM83A Y138 phosphorylation enhances Wnt/β-catenin signaling and pancreatic cancer cell proliferation and metastasis

**Fig. 6:**
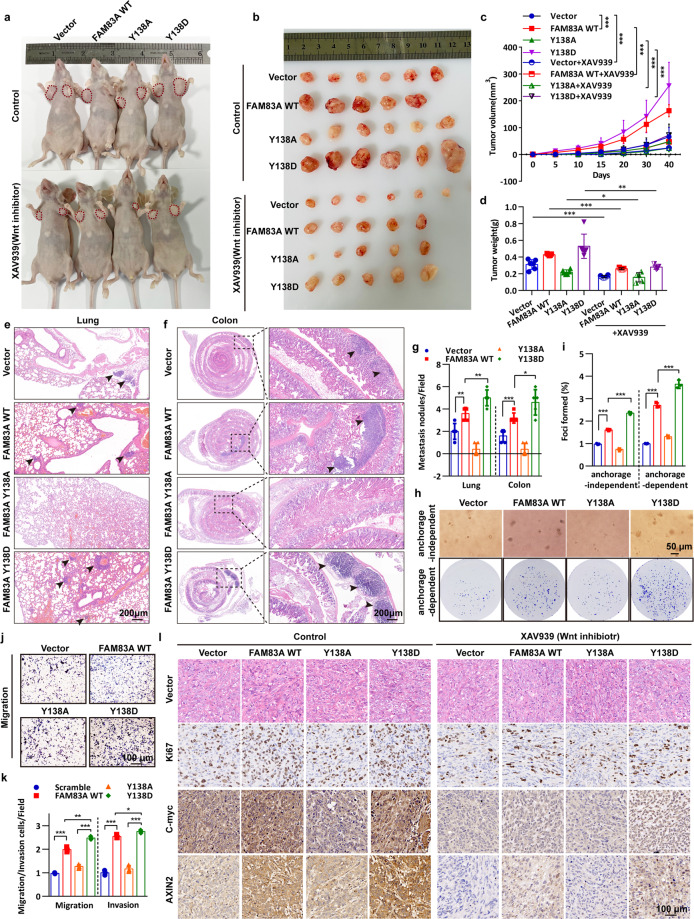
FAM83A Y138 phosphorylation enhances Wnt/β-catenin signaling and pancreatic cancer cell proliferation and metastasis. **a**, **b** Representative images of nude mice with two sides of axilla subcutaneous injected with PANC-1 cells stably expressed Flag-tagged wild-type FAM83A, Y138A, or Y138D mutants and representative images of excised tumors in different groups of nude mice were shown. The Wnt inhibitor XAV939 (20 mg/kg) was intravenously injected into the caudal vein of the nude mice one week later after xenograft formation. **c** Growth curve showing the changes in the tumor volume in mice in different groups from the injection (*n* = 6 or 5). **d** Weight of the excised tumors in each group (*n* = 6 or 5). **e**, **f** Representative images of lung and colon metastasis of the nude mice model that tail intravenous injected with PANC-1 cells stably expressed Flag-tagged wild-type FAM83A, Y138A, or Y138D mutants were shown. **g** The number of lung or colon metastasis was quantified per field (*n* = 5). **h**, **i** Representative images and quantification of the soft agarose and plate colony formation assays of PANC-1 cells stably expressed Flag-tagged wild-type FAM83A, Y138A, or Y138D mutants (*n* = 3). **j**, **k** Representative images and quantification of the transwell assays without matrigel of PANC-1 cells stably expressed Flag-tagged wild-type FAM83A, Y138A or Y138D mutants (*n* = 3). **l** Representative H&E staining images and immunohistochemical images of Ki67, C-myc, and AXIN2 expression in excised tumors tissues. *P < 0.05; **P < 0.01; ***P < 0.001. Data were presented as mean ± SD

We next explored the relevance of FAM83A phosphorylation in pancreatic cancer progression. To this end, we first generated PANC-1 cells harboring the wild-type FAM83A and its Y138A and Y138D mutants using the G418 screening system distinctive to puromycin in the FAM83A depletion background. In vivo proliferation and metastasis analysis indicated that PANC-1 cells harboring the FAM83A Y138D mutant showed elevated tumor proliferation and formed bigger xenograft tumors than the cells harboring the wild-type FAM83A in nude mice models (Fig. 6a–d). As expected, the Wnt signaling inhibitor XAV939 inhibits the xenograft tumor size mediated by FAM83A re-expression. Moreover, the FAM83A Y138D mutant showed much more lung and colon metastatic nodules (Fig. 6e–g). Consistently, FAM83A Y138D significantly enhanced anchorage-dependent and independent colony formation ability and cell proliferation in the in vitro assays (Fig. 6h, i). Furthermore, it also promoted pancreatic cancer cell migration and invasion activity (Fig. 6j, k, Supplementary Fig. S5a, b). In contrast, the FAM83A Y138A mutant restrained the tumorigenic potential induced by the wild-type FAM83A (Fig. 6a–k). It is also shown that FAM83A Y138 phosphorylation markedly increased the number of Ki67-positive cells compared with that in the control groups (Fig. 6l). To assess the alteration of Wnt signaling in the excised xenograft tumor tissues, immunohistochemical analysis of the Wnt target genes was performed, and the results showed that FAM83A phosphorylation significantly enhanced the levels of C-myc and AXIN2 (Fig. 6l). These results reveal that FAM83A Y138 phosphorylation enhances Wnt/β-catenin signaling and pancreatic cancer cell proliferation and metastasis.

### FAM83A is a direct downstream target of Wnt/β-catenin signaling and forms positive feedback in pancreatic cancer cells

**Fig. 7:**
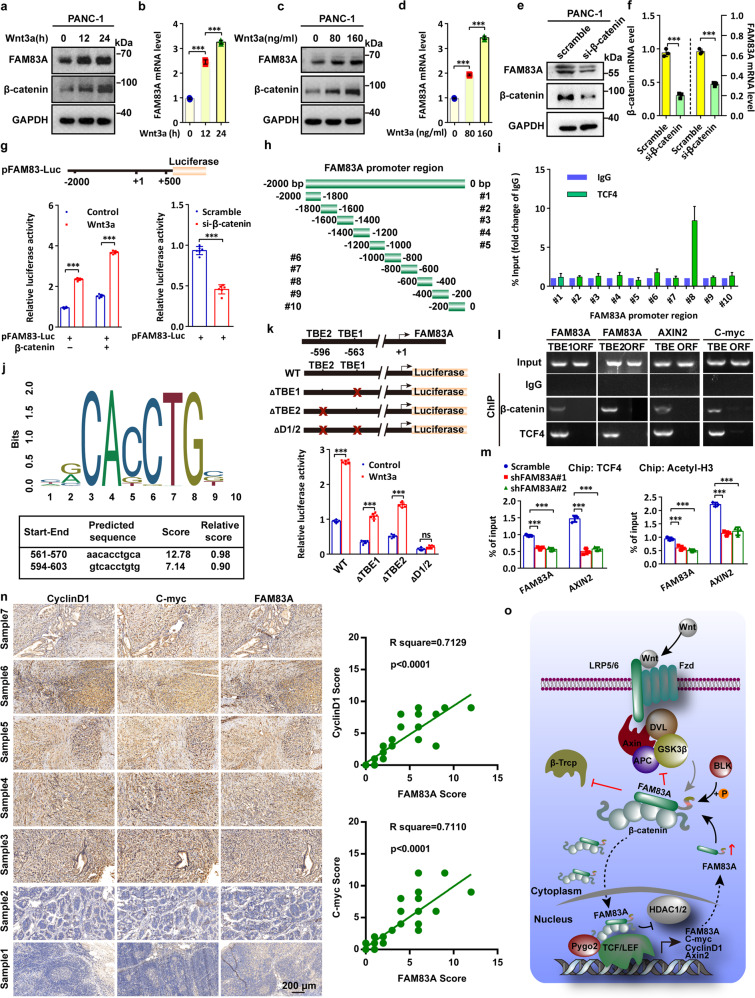
FAM83A is a direct downstream target of Wnt/β-catenin signaling and forms positive feedback in pancreatic cancer cells. **a**–**d** Representative western blotting images of the protein and mRNA levels of FAM83A after Wnt3a treatment for indicated concentrations and times in PANC-1 cells (*n* = 3). **e**, **f** The protein and mRNA levels of FAM83A after β-catenin knockdown in PANC-1 cells. Cell lysates were used for western blotting with the indicated antibodies (*n* = 3). **g** FAM83A gene promoter-reporter luciferase activity in HEK293T cells with or without β-catenin overexpression (left) and PANC-1 cells with or without β-catenin knockdown (right) (*n* = 6). **h** The schematic diagram of FAM83A gene promoter truncation and deletion generation. **i** PACN-1 cells were subjected to chromatin immunoprecipitation using antibodies against TCF4 and IgG, and the fold change of TCF/IgG was quantified (*n* = 3). **j** Predicted TCF4/LEF-binding sequence in the FAM83A gene promoter from the JASPAR website. **k** Schematic diagram of the generated luciferase report plasmids and the relative luciferase activity after transfection with the plasmids in HEK293T cells with or without Wnt3a treatment (*n* = 6). **l** A ChIP experiment was used to detect binding between the β-catenin-TCF4 complex and the predicted TCF/LEF-binding element in the FAM83A promoter (TBE1 and TBE2). C-myc and AXIN2 were used as a positive control (*n* = 3). **m** The level of FAM83A and Wnt target gene AXIN2 in the PANC-1 cells with FAM83A depletion in chromatin immunoprecipitation using TCF4 and histone H3 acetylation antibodies (*n* = 3). **n** Representative IHC images of FAM83A, CyclinD1, and C-myc in 44 human pancreatic cancer patient tissues. The correlation between the level of CyclinD1, C-myc, and FAM83A in 44 human pancreatic cancer patient tissues was shown. **o** Schematic diagram of the biological role of FAM83A in cytoplasmic β-catenin destruction complex and nuclear TCF4/β-catenin-mediated transcription in pancreatic cancer cells. *P < 0.05; **P < 0.01; ***P < 0.001. Data were presented as mean ± SD

As we noticed that Wnt3a treatment increased the mRNA level of FAM83A (Fig. 2g), we then asked whether a positive feedback loop might exist between Wnt/β-catenin signaling and FAM83A expression. The activation of Wnt/β-catenin signaling by Wnt3a elevated both the protein and mRNA levels of FAM83A in dose- and time-dependent manners (Fig. 7a–d), but β-catenin knockdown decreased FAM83A expression (Fig. 7e, f), suggesting that FAM83A is a target gene of Wnt/β-catenin signaling. By fusing the FAM83A promoter region (−2000 to +500) with a luciferase reporter sequence, we found that β-catenin overexpression promoted FAM83A luciferase reported activity with or without Wnt3a treatment, whereas β-catenin depletion showed the opposite effects (Fig. 7g). To assess the TCF4 binding elements of the FAM83A promoter region, we performed ChIP assays using a series of primers across 200 base pairs. The results showed that the −600 to −400 region of the FAM83A promoter contains the TCF4 binding sites (Fig. 7h, i). Surprisingly, it was predicted that there were two probable TCF/LEF-binding elements (TBE1 and 2) located on the −570 to −561 and −603 to −594 regions from the JASPAR website,^18^ which were contained in the TCF4 binding elements of the FAM83A promoter (Fig. 7j). Moreover, three luciferase reporters carrying the wild-type or the mutant FAM83A promoter regions were generated, and the results showed that deletion of both TBE1 and TBE2 completely abolished the reporter luciferase activity with or without Wnt3a stimulation (Fig. 7k). Furthermore, both β-catenin and TCF4 were found to occupy the region of the FAM83A promoter flanking these two TBE sites (Fig. 7l). Additionally, other ChIP assays with anti-TCF4 and anti-total acetylated histone H3 antibody demonstrated that FAM83A knockdown reduced the enrichment of TCF4 and histone H3 acetylation in the promoter region of FAM83A (Fig. 7m). We also confirmed a positive clinical correlation between FAM83A and Wnt/β-catenin target proteins in the tissues of 44 pancreatic cancer patients and the expression of FAM83A gene with Wnt/β-catenin genes from the TCGA database (Fig. 7n, Supplementary Fig. S5c). Furthermore, patients in the high FAM83A expression group exhibited shorter overall survival (OS) and worse disease-free survival (DFS) than those in the low expression group (Supplementary Fig. S5d). Together, these data indicate that FAM83A is a direct downstream target of Wnt/β-catenin signaling and forms positive feedback in pancreatic cancer cells (Fig. 7o).

### Peptides disrupting FAM83A-β-catenin interaction inhibit Wnt/β-catenin signaling

**Fig. 8:**
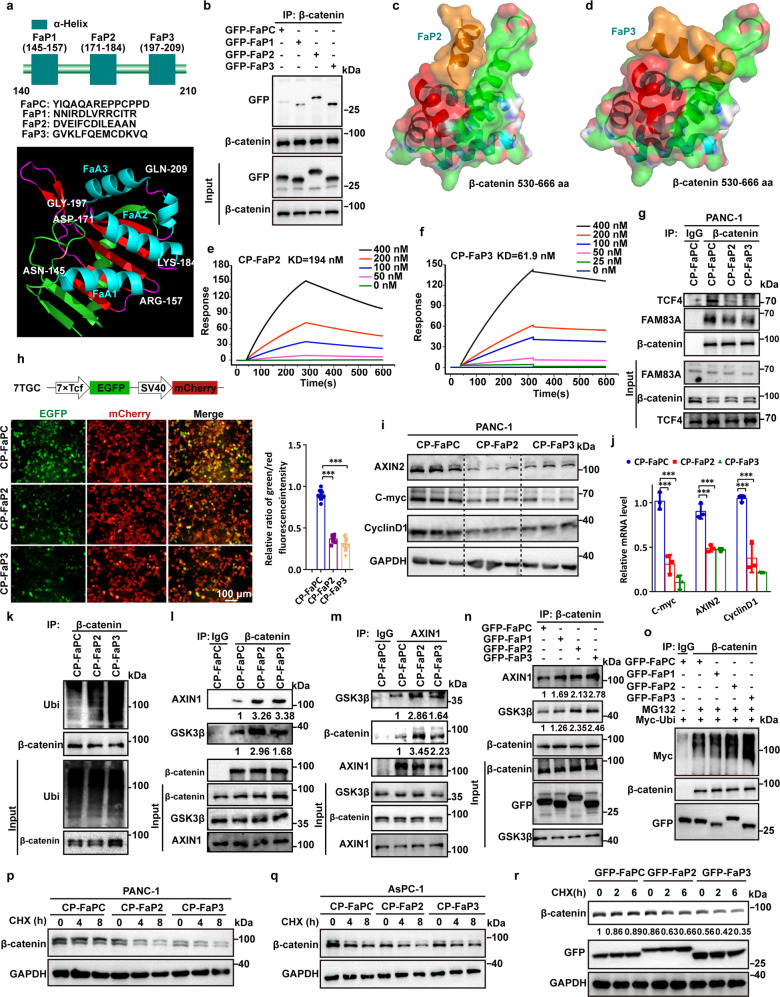
Peptides disrupting FAM83A-β-catenin interaction inhibit Wnt/β-catenin signaling. **a** Identification of β-catenin binding α-helical peptide(s) in FAM83A DUF1669 (140aa–210aa) region. The secondary structure of the FAM83A DUF1669 (140aa–210aa) region was analyzed based on the PDB data (4urj). **b** The FaPC, FaP1, FaP2, and FaP3 fragments were constructed into pEGFP-C1 expression vector connection with a flexible linker ((GGGGS)n). The interactions between β-catenin and the four fragments were evaluated with Co-IP assays in HEK293T cells (*n* = 3). **c**, **d** The predicted docking model of FaP2, FaP3 with β-catenin 530–666 aa region. **e**, **f** Kinetic interactions of cell-penetrating peptide TAT-linked peptides with β-catenin was determined by surface plasmon resonance analyses. **g** Effect of CP-FaP2 and CP-FaP3 on β-catenin-FAM83A and β-catenin-TCF4 interactions in PANC-1 cells. Cells were treated with control (CP-FaPC), CP-FaP2, and CP-FaP3 (5 μM) for 24 hours. Cell lysates were used for IP and western blotting with the indicated antibodies (*n* = 3). **h** The schematic diagram of 7TGC plasmid construction and the fluorescence images of PANC-1 cells with control, CP-FaP2, and CP-FaP3 treatment. The relative ratio of green/red fluorescence intensity was quantified (*n* = 9). **i**, **j** Protein and mRNA levels of Wnt target genes AXIN2, C-myc, and CyclinD1 in PANC-1 cells with control, CP-FaP2, and CP-FaP3 treatment (*n* = 3). **k** The level of β-catenin ubiquitination in PANC-1 cells after control, CP-FaP2 and CP-FaP3 treatment in PANC-1 cells (*n* = 3). Cell lysates were used for IP and western blotting with the indicated antibodies. **l**–**n** The interaction between β-catenin and AXIN1, GSK3β in PANC-1 cells after control, CP-FaP2 and CP-FaP3 treatment or GFP-tagged FaP1, FaP2, and FaP3 transfection in PANC-1 cells (*n* = 3). Cell lysates were used for IP and western blotting with the indicated antibodies. **o** The level of β-catenin ubiquitination in HEK293T cells after GFP-tagged FaP1, FaP2, and FaP3 transfection (*n* = 3). Cell lysates were used for IP and western blotting with the indicated antibodies. **p**–**r** The stability of β-catenin protein upon CHX treatment for indicated times in PANC-1 cells after control, CP-FaP2 and CP-FaP3 treatment or GFP-tagged FaP1, FaP2, and FaP3 transfection (*n* = 3). Cell lysates were used for IP and western blotting with the indicated antibodies

To verify the critical role of the FAM83A-β-catenin interaction in activating Wnt/β-catenin signaling and in pancreatic cancer tumorigenesis, we identified three α-helices derived from DUF1669 of the FAM83A protein based on the PDB data (4urj) (Fig. 8a). By fusing the three α-helical peptides with GFP via a flexible linker ((GGGGS)n), we found that FaP2 and FaP3 were mainly responsible for the FAM83A-β-catenin interaction compared with FaP1 and the control peptide (FaPC, a peptide that contains 13 amino acids from the DUF1669 domain distinguish from the region mediated FAM83A-β-catenin interaction) (Fig. 8b). Online predicted molecular docking results revealed that FaP2 and FaP3 bind to the groove formed by two α-helices of β-catenin Arm10–12, which mediated the interaction between FAM83A and β-catenin (Fig. 8c, d, Supplementary Fig. S6a). Cell-penetrating FaP2 (CP-FaP2), FaP3 (CP-FaP3), and FaPC (CP-FaPC) were then generated by linking a previously identified cell-penetrating peptide TAT.^19^ Both CP-FaP2 and CP-FaP3 showed an increased binding affinity with β-catenin as detected using surface plasmon resonance analysis (Fig. 8e, f). In vivo co-IP assays showed that CP-FaP2 and CP-FaP3 inhibit the interactions between FAM83A, TCF4 and β-catenin respectively compared with the control peptides (Fig. 8g). Moreover, the results showed that CP-FaP2 and CP-FaP3 inhibit β-catenin/TCF4-mediated transcriptional activity in the 7TGC transfected cells and suppress the mRNA and protein expressions of the Wnt/β-catenin genes C-myc, CyclinD1 and AXIN2 in both PANC-1 and AsPC-1 cells (Fig. 8h–j, Supplementary Fig. S6b–d). Consistently, the assembly of the β-catenin destruction complex and subsequent β-catenin ubiquitination were improved, whereas the stability of β-catenin was decreased upon CP-FaP2/CP-FaP3 treatment or GFP-FaP2/GFP-FaP3 transfection (Fig. 8k–o, Supplementary Fig. S6e, f). These data indicate that disrupting the FAM83A-β-catenin interaction by specific peptides inhibits Wnt/β-catenin signaling in pancreatic cancer cells.

### FAM83A-β-catenin interaction inhibitory peptides restrain pancreatic cancer development

**Fig. 9:**
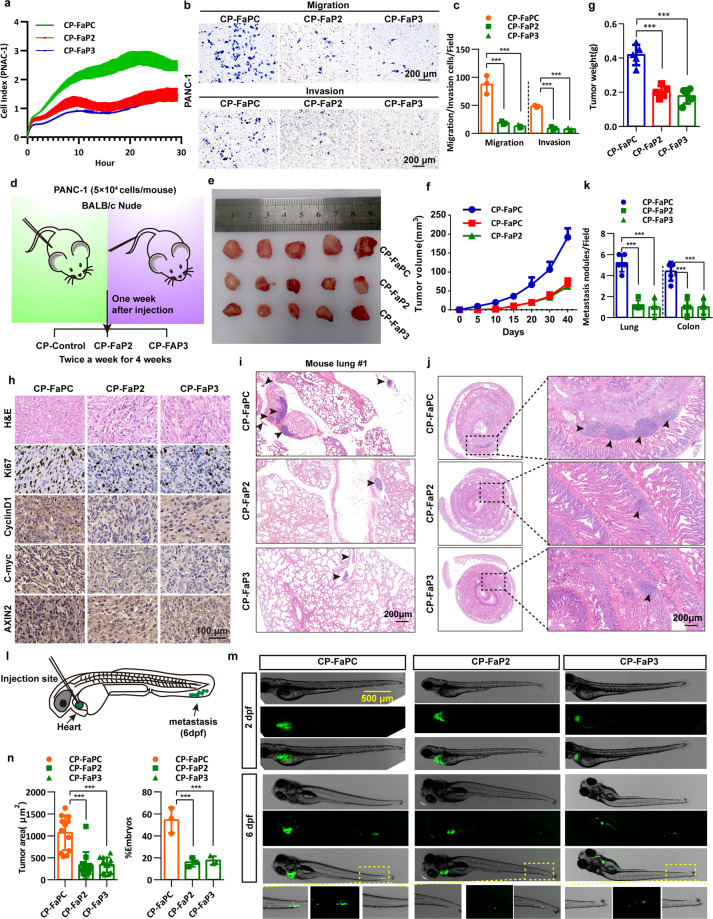
FAM83A-β-catenin interaction inhibitory peptides restrain pancreatic cancer development. **a** Real-time cell analysis (cell index, CI) showing the differences in growth profile for PANC-1 cells treated with control (CP-FaPC, 2 mg/kg/2d), CP-FaP2 (2 mg/kg/2d), and CP-FaP3 (2 mg/kg/2d) peptides measured in a 16-well E-plate. **b**, **c** Representative images and quantification of the transwell assays with or without matrigel of PANC-1 cells treated with control, CP-FaP2, and CP-FaP3 peptides (*n* = 3). **d** Schematic diagram of the experimental procedure of the treatment of the peptides to nude mice xenograft formation and tail vein injection. **e** Representative images of excised tumors in the groups with respective peptides treatment were shown. **f** The growth curve shows the changes in the tumor volume in mice in different groups (*n* = 5). **g** Weight of the excised tumors in each group (*n* = 5). **h** Representative H&E staining images and immunohistochemical images of Ki67, C-myc CyclinD1, and AXIN2 expression in excised tumors tissues. **i**–**k** Representative images and quantification of lung and colon metastasis of each group was shown (*n* = 5). **l** Schematic diagram of the zebrafish embryo xenograft assay. **m** Representative overview images at two days and six days post-implantation of zebrafish injected with EGFP-tagged PANC-1 cells. Scale bar: 500 μm. **n** Quantification of the area of metastatic cells in individual zebrafish embryos (*n* = 15) and the percentage of the zebrafish embryos displaying tumor progression to the tail fin (*n* = 3)

To examine the effects of the peptides that target FAM83A-β-catenin interaction on the tumorigenicity of pancreatic cancer cells, in vitro and in vivo assays were performed, and the results showed that CP-FaP2 and CP-FaP3 decreased the ability of DNA synthesis of PANC-1 and AsPC-1 cells compared with that observed in the control cells by using a 5-ethynyl-20-deoxyuridine (EdU) incorporation assay (Supplementary Fig. S7a–d). Impedance-based real-time cell analysis (RTCA) showed that the recorded cell index (CI), which correlated with the reported cell proliferation potential of the cancer cells, significantly decreased in the CP-FaP2 and CP-FaP3 treated PANC-1 cells (Fig. 9a). Furthermore, we also found that CP-FaP2 and CP-FaP3 dramatically inhibited anchorage-dependent or anchorage-independent cell growth judged by foci formation or soft agar assay in both PANC-1 and AsPC-1 cells (Supplementary Fig. S7e–h). Furthermore, results of the wound healing migration, transwell migration, and matrigel invasion assays revealed that CP-FaP2 and CP-FaP3 significantly reduced the motility of pancreatic cancer cells (Fig. 9b, c, Supplementary Fig. S7i–n).

We next generated xenograft tumor models by subcutaneously injecting PANC-1 cells into the hind limbs of nude mice and a lung and colon metastasis mouse model by tail vein injection of the PANC-1 cells (Fig. 9d). Consistent with the in vitro results, CP-FaP2 and CP-FaP3 suppressed xenograft tumor growth and decreased the expression of Ki67 and Wnt/β-catenin target genes in inoculated tumor tissues compared with that of the control peptides (Fig. 9e–h). Moreover, fewer and smaller lung and colon metastatic nodules were observed in mice treated with CP-FaP2 and CP-FaP3 peptides compared to those in the control group (Fig. 9i–k). Furthermore, we tested the role of CP-FaP2 and CP-FaP3 in malignant pancreatic cancer by using a zebrafish embryo xenograft model, in which injected fluorescence-labeled tumor cells can metastasize into the tail fins (Fig. 9l). The results revealed that the embryos transplanted into pancreatic cancer PANC-1 cells treated with CP-FaP2 or CP-FaP3 showed much less tumor progression than the embryos treated with the control peptides (Fig. 9m). Both the average invasive and the percentages of embryos exhibiting metastasis were significantly lower in the two groups treated with CP-FaP2 or CP-FaP3 (Fig. 9l). Taken together, these data indicate that disrupting the FAM83A-β-catenin interaction inhibits pancreatic cancer development.

## Discussion

Recent studies have demonstrated that FAM83A is significantly upregulated in a variety of cancers and is presumed to be a novel oncogene partly through activating the PI3K/AKT and Wnt signaling cascade pathway.^10–15^ However, the detailed mechanisms of FAM83A in cancer, especially in pancreatic cancer, remain extremely unclear. In the present study, we first reported that FAM83A is a β-catenin binding patterner through the DUF1669 domain and plays an important role in the assembly of the destruction complex and subsequent β-catenin protein ubiquitination and stability. We also demonstrated that FAM83A significantly shifts from the cytoplasm to the nucleus upon Wnt activation and inhibits HDAC recruitment by TCF4, thus altering the histone acetylation modification of the Wnt/β-catenin target genes. Moreover, we recognized that some of the SRC or TEC non-receptor kinase subfamily members, such as LYN and BLK, could bind and phosphorylate FAM83A at the tyrosine residue(s). The phosphorylation states of FAM83A at tyrosine 138 residue are shown to be essential for the activation of Wnt/β-catenin signaling and the development of pancreatic cancer.

High-frequency somatic mutations in a subset of genes and constitutively activated signaling pathways, such as the KRAS (KRAS proto-oncogene) and Wnt signaling pathways, are considered to be the main cause of pancreatic cancer development.^4^ The DUF1669 domain, which is conservative among the FAM83 members, mediates the signal cascade of the EGFR/RAS/CRAF pathway and EGFR- and RAS-driven oncogenic transformation.^20^ Consistently, FAM83A is identified using a retroviral cDNA library screen that confers resistance on an EGFR-tyrosine kinase inhibitor in tumorigenic mammary epithelial cells.^9^ Moreover, recent observations confirm that FAM83 members A-E can independently promote anchorage-independent growth and form complexes with CRAF in mammary epithelial cells.^21^ Research using mouse models of pancreatic cancer or other cancers has similarly reported nodes of crosstalk between Wnt signaling networks and RAS oncogene. Overexpression of constitutively active β-catenin produces no lung tumor, whereas co-expression of constitutively active forms of both KRAS and β-catenin induces lung tumors, as shown in one model of lung cancer.^22^ Expression of constitutively active β-catenin also synergizes with the active forms of NRAS to induce melanomas with short latency.^23^ Inherited inactivating mutations in Wnt signaling negative regulator APC are found in patients with familial adenomatous polyposis (FAP), which can progress to colorectal carcinomas following concomitant activating mutations in KRAS and inactivating mutations in TP53.^24^ Here, our results revealed positive feedback that the Wnt signaling factor TCF4 binds to the promoter region of FAM83A and initiates its transcription. Our finding also proposes an approach that mediates the interplay between Wnt signaling and RAS cascade activation in the development of pancreatic cancer. Previous reports have shown that the FAM83 protein family has been annotated as containing a putative phospholipase D-like (PLD-like, HxKxxxxD) motif, which lacks conservation at a critical histidine residue that is present in all bona fide PLD enzymes for catalytic activity.^25^ Both FAM83A and FAM83B were unable to exhibit phospholipase activity.^9,20^ However, our data show that the domain aa 150–250, which mediated the interaction between FAM83A and β-catenin, exactly coincided with the PKD-like motif. Hence, future studies are needed to assess the features within the DUF1669 domain that still harbor certain pseudo-PLD roles, such as binding to specific phospholipids, that might affect binding to β-catenin.

Macroautophagy (hereafter referred to as autophagy) is a process in which organelles termed autophagosomes deliver cytoplasmic constituents to lysosomes for degradation.^26^ Autophagy is essential for the development of pancreatic cancer.^27,28^ It was reported that the Wnt/β-catenin signaling activity directly repressed the transcription of SQSTM1 via TCF4 and suppressed autophagosome formation.^29^ β-catenin and Disheveled (Disheveled segment polarity proteins) could be selectively degraded and thus showing inhibited Wnt signaling via the formation of an LC3 or SQSTM1 binding complex during nutrient deprivation.^30,31^ RACK1 was reported to promote autophagy by enhancing the ATG14-Beclin1-VPS34-VPS15 complex formation^32^ but suppresses Wnt signaling and gastric tumorigenesis by stabilizing the β-catenin destruction complex.^33^ However, it is still largely unknown the mechanisms of the existence of high levels of both autophagy and Wnt/β-catenin signaling in pancreatic cancer. In our previous study, we demonstrated that lncRNA PVT1 up-regulates the expression of both Pygo2 and ATG14 and thus regulated Wnt/β-catenin signaling and autophagic activity to overcome gemcitabine resistance through sponging miR-619-5p.^34^ The positive autophagy initiation factor STYK1 probably enhances Wnt/β-catenin signaling through the AKT/GSK3β cascade.^35–37^ Moreover, we also showed that the β-catenin/TCF4 complex elevates the mRNA and protein levels of TSPAN1, a positive regulatory protein that functions in autophagy maturation, through binding directly to the two conservative TCF/LEF-binding elements in the promoter region of the TSPAN1. Furthermore, the upstream microRNA that targets both TSPAN1 and FAM83A is significantly downregulated.^2^ Here, our data detailed the mechanisms of FAM83A that promote Wnt/β-catenin signaling and together with our previous report defined the crosstalk between autophagy and Wnt in pancreatic cancer development.

Intricate communication between pancreatic cancer cells and their surrounding environment is driven by a dynamic signaling network of cellular and matrix remodeling enzymes, cytokines, chemokines, and growth factors, which collectively promote tumor growth and treatment resistance. The non-receptor SRC kinase family signaling network is increasingly recognized as a key player in pancreatic tumorigenesis.^38,39^ Treatment of primary PDAC cultures established from patient-derived xenografts with dasatinib or PP2 reduced the clonogenic, self-renewal, and tumor-initiating capacity of pancreatic cancer stem cells (PaCSCs). Insulin receptor 1, which plays a role in GLUT4 and GLUT1 translocation, was reported to be phosphorylated by LCK/YES-related novel kinase (LYN) directly and appears to be required for oncometabolite changes.^40^ The inhibition of SRC family kinase YES1, LYN, FYN, FRK, and C-SRC using specific small interfering RNA transfection markedly suppressed cell proliferation in PANC-1 cells.^41^ The recent identification of BLK as a player in the differential phosphorylation signatures of PDAC cells suggests that BLK may play an important role in solid pancreatic malignancies.^42^ Increased BLK activity was also reported to lead to increased PDX1 activation and enhanced insulin synthesis, which is being investigated as a potential target for pancreatic cancer therapy.^43,44^ Consistently, our data also show that FAM83A could be phosphorylated by the non-receptor SRC kinase subfamily members, in which BLK kinase presents central effects. Moreover, the phosphorylation states of FAM83A revealed an oncogenic function and presented a link between Wnt/β-catenin signaling and BLK kinase in the progress of pancreatic cancer. Although the conserved PxxP motif was predicted to interact with Src homology 3 (SH3) domain-containing proteins in the PRD domain of FAM83A,^45^ our results reveal that the DUF1669 domain-mediated binding of FAM83A to BLK kinase.

The β-catenin transcriptional complex is a high-priority pharmacologic target because of its pathologic role in a broad range of human cancers. Indeed, the chimeric peptide CP-FaP2 or CP-FaP3 interferes with the formation of the FAM83A-β-catenin and β-TCF4 complex, decreasing the stability of β-catenin, and counteracts the oncogenic capability of pancreatic cancer cells in vitro and in vivo. Our data also show that the peptide dosing interval of twice a week resulted in an ideal treatment outcome. In summary, our results detail the roles of FAM83A in the regulation of Wnt/β-catenin signaling through directly binding to β-catenin and suppressing TCF4-mediated transcriptional activity. In addition, our results provide a proof of concept for a therapeutic strategy against pancreatic cancer development.

## Materials and methods

### Mouse subcutaneous xenograft and metastasis experiments

All animal experiments were carried out in compliance with a protocol specifically approved for the use of laboratory animals by the Hubei University of Technology Animal Care and Use Committee. Four-week-old (18–22 g) female BALB/c nude mice were purchased from Hunan SJA laboratory animal (Changsha, China). PANC-1 cells (3 × 10^6^) were subcutaneously injected into the left and right axillae, or into the tail vein of five female BALB/c nude mice per group for the xenograft model and lung/colon metastasis model, respectively. One or two weeks later, the animals were treated with Wnt inhibitor XAV939 at 50 mg/kg body weight or commercially synthetic peptides at 2 mg/kg body weight via intraperitoneal injection twice a week. The length and width of the mouse xenograft tumors were measured every 5 days with calipers, and tumor volume (V) was calculated with the following formula: *V* = [(length × width × width)/2]. Experimental mice were euthanized at the end of the observation period, and then the tumors were excised and weighed. The metastasis of tumor cells to the lung or colon was also quantified after euthanasia. The subcutaneous xenograft and metastasis mouse models were used to assess the tumor formation and metastasis ability of pancreatic cancer PANC-1 cells, which were stably expressed wild-type FAM83A, FAM83A Y138A. and FAM83A Y138D mutants with or without XAV939 or functional peptides treatment.

### Cell lines and reagents

Human pancreatic cancer cell lines PANC-1 (TCHu 98), AsPC-1 (TCHu 8), SW1990 (TCHu201), and CFPAC-1 (TCHu112) were purchased from the cell center of the Institute of Biochemistry and Cell Biology or National Collection of Authenticated Cell Cultures, Chinese Academy of Sciences (Shanghai, China). HEK293T and HeLa cells were stored in our lab. HEK293T, HeLa, PANC-1, and AsPC-1 cells were cultured in Dulbecco’s modified Eagle’s medium (12500096, Gibco, USA). All culture mediums were supplemented with 10% fetal bovine serum (G10270-106, Gibco, USA), 100 U/ml penicillin G, and 100 μg/ml streptomycin (P1400, Solarbio) at 37 °C in a humidified incubator containing 5% CO_2_. The medium was replaced every 2–3 days and the cell was subcultured and used for an experiment at 80–90% confluence. Wnt agonist Wnt3a was purchased from Abcam (ab153563). Wnt inhibitor XAV939 was purchased from MedChemExpress (HY-15147). BLK kinase inhibitor saracatinib and LCK kinase inhibitor PP2 were both purchased from MedChemExpress (HY-10234 and HY-13805). Cycloheximide (CHX) and MG132 were purchased from Selleck (S7418 and S2619).

### Western blotting and immunoprecipitation

Immunoprecipitation and Western blotting were performed as described previously.^35^ GAPDH protein levels were used as loading controls. We used the following primary antibodies: anti-Non-phospho (Active) β-catenin (Ser33/37/Thr41) (8814, Cell Signaling Technology), anti-phospho-β-catenin (Ser33/37/Thr41) (9561, Cell Signaling Technology), anti-β-catenin (51067-2-AP, Proteintech), anti-β-TrCP (4394, Cell Signaling Technology), anti-FAM83A (A15201, ABclonal), anti-c-myc (10828-1-AP, Proteintech), anti-CyclinD1 (60186-1-Ig, Proteintech), anti-AXIN2 (20540-1-AP, Proteintech), anti-mouse HA (M180-3, EMD Millipore), anti-Rabbit HA (51064-2-AP, Proteintech), anti-mouse DYKDDDDK (M185, MBL), anti-Rabbit DYKDDDDK (80010-1-RR, Proteintech), anti-GAPDH (60004-1-Ig, Proteintech), anti-GFP (598, EMD Millipore), anti-phosphotyrosine (P4110, Sigma), anti-phosphoserine (05-1000x, EMD Millipore), anti-phospho-threonine (9386 S, Cell Signaling Technology), anti-GSK3β (22104-1-AP, Proteintech), anti-AXIN1 (16541-1-AP, Proteintech), anti-TCF4 (22337-1-AP, Proteintech), anti-BLK (10510-1-AP, Proteintech), anti-HDAC1 (10197-1-AP, Proteintech), anti-HDAC2 (12922-2-AP, Proteintech), anti- Acetyl-Histone H3 (8173, Cell Signaling Technology).

### Lentiviral production and creation of stable cell lines

Lentiviral production was performed as described previously.^34^ Briefly, FAM83A shRNAs or scramble RNA were subcloned into the lentiviral vector pLKO.1-puro (Sigma, 8453). DNA fragments encoding FAM83A and its Y138A or Y138D mutants were subcloned into lentiviruses vector pCDH-CMV-MCS-EF1-turboRFP-T2A-Neo. 5 μg lentiviral constructs were co-transfected with viral packaging plasmids 3 μg psPAX2 and 3 μg pMD2.G into 293 T cells in 10 cm dishes for the lentiviral particle production. The viral supernatant was harvested at 48 h post-transfection and filtered through a 0.22 µm membrane. After applying the viral supernatant to PANC-1 cells with 10 μg/μl of polybrene (Solarbio, H8761), selection for puromycin and/or G418 resistance was initiated 48 hours after transfection. The selection media was changed every 3-4 days for several weeks, and clones of puromycin and/or G418 - resistant cells were isolated and expanded for further characterization. The stable cells were maintained with a complete culture medium with 2 μg/ml puromycin and/or 100 μg/ml G418.

### Flag-tag affinity/LS-MS/MS analysis

The HEK293T cells cultured in 10 cm dishes were transiently transfected with an empty vector or Flag-FAM83A for 48 h. The cells were collected and washed with cold PBS and lysed in lysis buffer supplemented with protease and phosphatase inhibitors. The cell lysates were subjected to IP assay using the Flag antibody, and the immunoprecipitate was sent for LS-MS/MS analysis.

### In vitro tyrosine kinase activity assay

The lysates of HEK293T cells were co-incubated with purified GST-tagged FAM83A with or without ATP addition. Then the products were sent to western blotting assays using an anti-phosphotyrosine antibody.

### Dual-luciferase reporter assay

The FAM83A promoter region which from 2000 bp upstream to 500 bp downstream of the transcription start site was cloned into the firefly luciferase reporter plasmid pGL3-Basic (Promega, E1751). The renilla luciferase vector was used as an internal control and was purchased from Promega (Promega, E6971). The primer sequences used for FAM83A promoter qRT-PCR primer sequences were (forward) CACACAGAGATATGTGTGTGTGTATA and (reverse) GCCAGTGAGCTGGGAACTG. The nucleotide sequences of all constructs were confirmed by DNA sequencing. The site mutations or depletions of the FAM83A promoter region luciferase reporter construction were generated using the overlap extension PCR assay or QuikChange II XL Site-Directed Mutagenesis Kit (Stratagene, 200521). The luminescence ratio of the experimental reporter (Firefly) to the control reporter (Renilla) was calculated. All experiments were performed in triplicate.

### Zebrafish maintenance, embryo preparation, and tumor cell implantation

Zebrafish and embryos were raised, staged, and maintained according to standard procedures. All zebrafish experiments were approved by the Hubei University of Technology Animal Care and Use Committee. Dechorionized 2 days post fertilization (dpf) zebrafish embryos were anesthetized with 0.003% tricaine (Sigma, E10521), and then positioned on a 10 cm Petri dish coated with 3% agarose. Pancreatic cancer PANC-1 cell suspensions labeled with GFP were loaded into borosilicate glass capillary needles and the injections were performed using a pneumatic pico-pump and a manipulator (WPI, Stevenage, UK). Approximately 500 cells were injected at ~60 μm above the ventral end of the duct of Cuvier (DoC), where the DoC opens into the heart. Then the zebrafish embryos were maintained at 33 °C and photographed under an Olympus FSX100 microscope.

### Statistical analysis

All experiments were performed independently at least three times. All statistical analyses were performed using GraphPad Prism 6.0 software (GraphPad, La Jolla, CA, USA). All data are presented as the mean ± SD (standard deviation) from triplicates. Differences with a *p* value <0.05 were statistically significant. Differences between two groups were analyzed by independent sample t-tests and differences among multiple groups by one-way ANOVA; *represents *P* < 0.05, **represents *P* < 0.01, and ***represents *P* < 0.001.

## Supplementary information

Supplementary Material

## Data Availability

All data generated or analyzed during this study are included in the article.
