# Supplementary material for: B-lymphoid tyrosine kinase-mediated FAM83A phosphorylation elevates pancreatic tumorigenesis through interacting with β-catenin
Source: Signal Transduct Target Ther. 2023 Feb 17;8:66. doi: 10.1038/s41392-022-01268-5 (PMC9935901; doi:10.1038/s41392-022-01268-5)
Supplement: Supplementary file 1 — Supplementary Material [file 41392_2022_1268_MOESM1_ESM.docx]

Supplementary Materials for

B-lymphoid tyrosine kinase mediated FAM83A phosphorylation elevates pancreatic tumorigenesis through interacting with β-catenin

Cefan Zhou^1, 2 #^, Xiaoting Zhu^1#^, Nanxi Liu^1, 3#^, Xueying Dong^1^, Xuewen Zhang^1^, Huili Huang^1,4^, Yu Tang^1^, Shicheng Liu^1^, Mengyu Hu^1^, Ming Wang^5^, Xiaoling Deng^1^, Shi Li^1^, Rui Zhang^1^, Yuan Huang^1^, Hao Lyu^1^, Shuai Xiao^1^, Sang Luo^6^, Declan William Ali^7^, Marek Michalak^8^, Xing-Zhen Chen^2^, Zhentian Wang^4*^ and Jingfeng Tang^1*^

Correspondence to: [Jingfeng_hut@163.com](mailto:Jingfeng_hut@163.com) and [zhentian@fudan.edu.cn](mailto:zhentian@fudan.edu.cn)

**This PDF file includes:**

Materials and Methods

Figures. S1 to S7

**Materials and methods**

**Plasmids, siRNAs and transfection**

DNA fragments encoding BLK, SRC, BTK, ITK, TEC, LYN, FYN, FRK, LCK, HCK and YES1 amplified by PCR were gifts from Prof. Jiahuai Han (Xiamen University, China) and cloned into pcDNA3.0-3×HA. The PCR products of FAM83A, GSK3β and AXIN1 were cloned into pcDNA3.0-3×HA, pCMV-3×Flag (Sigma, E4401) and pCMV-mCherry (constructed by our lab) respectively. The PCR products of β-catenin were cloned into pCMV-N1-GFP (Clontech, 6085-1). To make FAM83A or BLK mutants, a series of forwarding primers harboring the desired mutations were used in PCRs to generate the FAM83A Y138A, Y138D and BLK Y389A mutations. To make deletion mutations, overlap extension PCR was used to generate DNA sequences encoding FAM83A 1-50, 51-100, 101-150, 151-200, 201-250, 251-296, 1-296, 297-434, Δ1-100, Δ101-200, Δ201-296, β-catenin 1-150, 151-666, 667-781, 151-276, 277-389, 400-530, 531-666. GSK3β 1-123, 1-353. The nucleotide sequences of all constructs were confirmed by DNA sequencing. 7TGC was a gift from RoelNusse (Addgene, 24304). DNA and interference RNA were transfected with Lipofectamine 3000 (Invitrogen, L3000015) according to the manufacturer’s instructions. shRNA for FAM83A #1 was 5ʹ- GCACAACAACATCAGAGACCTCTCGAGAGGTCTCTGATGTTGTTGTGC -3ʹ. shRNA for FAM83A #2 was 5ʹ- CTCTGACAGTCACCTCAAGAACTCGAGTTCTTGAGGTGACTGTCAGAG -3ʹ. siRNA for BLK #1 was 5ʹ- AGAUGAAGGGAGCAGAUUGUC -3ʹ, siRNA for BLK #2 was 5ʹ- GCCGAUCAAAGAGAAGGACAA -3ʹ. siRNA for β-catenin was 5ʹ- AGGUGCUAUCUGUCUGCUCUA -3ʹ.

**RNA extraction and qRT-PCR**

Total RNA was extracted from cultured pancreatic cancer cells using Trizol reagent (Invitrogen) according to the manufacturer’s protocol. qRT-PCR was performed as previously described.[^1^](#_ENREF_1) The mRNA expression level for each sample was normalized to the expression of GAPDH using the 2 ^-ΔΔct^ method[^2^](#_ENREF_2) with three biological replicates of comparative qRT-PCR. The following primer sequences were used for qRT-PCR: GAPDH, (forward) 5′- AGCCACATCGCTCAGACAC -3′ and (reverse) 5′- GCCCAATACGACCAAATCC -3′; CyclinD1, (forward) 5′- GCGTGTAGCTATGGAAGTTGCA -3′ and (reverse) 5′- CATCCCGAATGAGAGTCCTACAG -3′; C-myc, (forward) 5′- ATCTCACAGTGACCAACCCAAA -3′ and (reverse) 5′- TCGGTCACGGAGCCAATC -3′; AXIN2, (forward) 5′- CAAGGGCCAGGTCACCAA -3′ and (reverse) 5′- CCCCAACCCATCTTCGT -3′; FAM83A, (forward) 5′- CGGAAGCAGGCCCTAAGG -3′ and (reverse) 5′- CCTCTAGAATGTCACAGAAGATCTCC -3′. Amplification was performed in an ABI Q3 real-time PCR system using a SYBR Green master mix (Invitrogen, USA). The mixture was preheated for 10 min at 95 °C, followed by 40 cycles of amplification (30 s at 95 °C and 1 min at 58 °C, respectively). The Ct value of each sample was calculated and the relative mRNA expression level was normalized to that of GAPDH.

**TOP/FOP ﬂash assay**

M50 Super 8x TOPFlash and M51 Super 8x FOPFlash (TOPFlash mutant) were gifts from Randall Moon (Addgene, 12456 and 12457). The human pancreatic cancer PANC-1 or HEK293T cells were transfected with the M50 and M51plasmids along with an internal Renilla control plasmid. The luminescence ratio of the experimental reporter (Firefly) to the control reporter (Renilla) was calculated. All experiments were performed in triplicate.

**Glutathione S-Transferase pulldown**

Purified His-tagged FAM83A protein was incubated with GST or GST-tagged FAM83A fusion protein and glutathione-sepharose beads overnight at 4°C. Expression of GST fusion proteins were confirmed by western blotting assay.

**Chromatin immunoprecipitation assay (ChIP)**

Chromatin immunoprecipitation assay (ChIP) was performed as described previously.[^3^](#_ENREF_3) Briefly, cross-linked chromatin and protein complex using 1% formaldehyde was sonicated into ~500 bp fragments. The soluble material was then purified by centrifugation and mixed with the polyclonal antibodies against TCF4, β-catenin, Acetyl-H3 or control rabbit IgG overnight at 4 °C, followed by magic protein A/G beads under reverse rotation at 4 °C for 2 hours. qPCR was used to analyze the binding of the β-catenin /TCF-4 complex to the FAM83A promoter. The primers are listed below: #1 (-1800~-2000) (forward), 5′- cacacagagatatgtgtgtgtgta -3′ (reverse), 5′- aatggacttattagatatgaattggaga -3′; #2 (-1600~-1800) (forward), 5′- tcagtttcccccttttctc -3′ (reverse), 5′- cccaaacagggcaaaga -3′; #3 (-1400~-1600) (forward), 5′- ctctgactggcccct -3′ (reverse), 5′- cctctcttttcttctggaaca -3′; #4 (-1200~-1400) (forward), 5′- ggaggtgtggagcagg -3′ (reverse), 5′- tggtcagtgaaccaacctt -3′; #5 (-1000~-1200) (forward), 5′- cccagtgcagcgc -3′ (reverse), 5′- ttcctcatccctatagtaacatttg -3′; #6 (-800~-1000) (forward), 5′- agtgaagttcagtaaggtcc -3′ (reverse), 5′- aaggcgcttgtagaatatgt -3′; #7 (-600~-800) (forward), 5′- gcggaccagaagagg -3′ (reverse), 5′- gacgggccctcacc -3′; #8 (-400~-600) (forward), 5′- acctgtgagtgtgttcc -3′ (reverse), 5′- acccccatccaggg -3′; #9 (-200~-400) (forward), 5′- ccaaaatcatctccagctga -3′ (reverse), 5′- cactgtggattatggaggac -3′; #10 (0~-200) (forward), 5′- cctgtcactgagggc -3′ (reverse) 5′- aaactggttggtgattctcatc -3′; AXIN2-TBE, (forward) 5′- TCTTGCCTTCCTCTCACTT -3′, (reverse), 5′- GCTCATCTGAACCTCCTCTCT -3′; AXIN2-ORF, (forward) 5′- AGGCCCGCTCGGATCTTTT -3′, (reverse), 5′- GGGGAACATGGGGAGTCGTG -3′; C-myc-TBE, (forward) 5′- GCTCAGTCTTTGCCCCTTTGTGG -3′, (reverse), 5′- TAACACCTTCCCGATTCCCAAGTG -3′; C-myc-ORF, (forward) 5′- cagagaagctggcctcc -3′, (reverse), 5′- ctggagtcttgcgaggc -3′.

**Analysis of Surface Plasmon Resonance**

Binding kinetics between β-catenin and commercially synthetic α-helical peptides were measured by surface plasmon resonance using a Biacore T200 instrument (GE Healthcare, Chicago, IL).[^4^](#_ENREF_4) The dissociation constant was calculated according to BIA evaluation software (GE Healthcare).

**Immunohistochemistry**

Immunohistochemistry was performed as described previously.[^1^](#_ENREF_1) Briefly, the antibody against Ki67, CyclinD1, C-myc and AXIN2 were tested on sections from excised xenograft tumor tissues.

**Impedance-based real-time cell analysis (RTCA)**

The rate of cell proliferation was monitored in real time using the xCELLigence system (E-plate and E-insert). Totally 4×10^4^ PANC-1 cells were seeded in the E-plate. Data were collected and the impedance value was automatically monitored by the xCELLigence system for indicated times and expressed as a Cell index (CI) value.

**Cell viability assay**

Cell viability was determined by MTT assay which was performed as described previously.[^5^](#_ENREF_5) Briefly, PANC-1 cells were seeded into 96 - well plates. After 12 h culture, the cells were treated with commercially synthetic peptides with indicated concentration for another 24 h. Cells were then stained with 100 μl sterile MTT dye (0.5 mg/ml; Sigma, M2128) for 4 h at 37°C, followed by removal of the culture medium and the addition of 150μl DMSO (Sigma, W387520). The number of viable cells was assessed by measurement of the absorbance at 450 nm by a microplate reader. All experiments were performed in triplicate. For calculation of half inhibition concentration (IC50), data were fitted in Graph Pad Prism 6.0 and the dose-response curve was plotted using the equation log (inhibitor) vs. response- Variable slope. The IC50 was obtained using formula: Y=Bottom + (TopBottom)/ (1+10^ ((Log IC50-X) *HillSlope)).

**Colony formation assay**

For plate colony formation assay, human pancreatic cancer PANC-1 and AsPC-1 cells stably expressing wild-type FAM83A and its Y138A, Y138D mutants were seeded into 24 - well plate and incubated with complete medium at 37 °C for 2 - 3 weeks. Then, the cells were fixed with 4% paraformaldehyde and stained with 2% crystal violet. The images were obtained and the number of colonies was counted. For soft agar colony assay, PANC-1 or AsPC-1 cells were mixed with 0.3% agarose and plated onto a 0.5% agarose underlay. After culture for two weeks at 37 °C in a 5% CO2 incubator, the number of colonies >100 μm was counted. Each experiment was repeated three times.

**5-Ethynyl-20-deoxyuridine (EdU) incorporation assay**

EdU labeled PANC-1 and AsPC-1 cells with or without stably expressing wild-type FAM83A and its Y138A or Y138D mutants were examined with the BeyoClick™ EdU Cell Proliferation Kit with Alexa Fluor 555 or 488 (Beyotime, C0075S, C0071S). Cells were photographed under an Olympus FSX100 microscope.

**Wound-healing assay**

PANC-1 and AsPC-1 cells stably expressing wild-type FAM83A and its Y138A, Y138D mutants were placed in twelve - well plates and grown until 90% confluence. A straight wound through the cell layer was made with a sterile pipette tip. Then, cells were treated with control, CP-FaP2 and CP-FaP3 peptides and incubated for 24 h. Cell migration was analyzed via the wound closure ratio.

**Trans-well assays**

PANC-1 and AsPC-1 cells stably expressing wild-type FAM83A and its Y138A, Y138D mutants were placed in the upper well of an uncoated trans-well chamber with an 8-µm φ pore membrane. Then, DMEM culture medium containing 20% FBS was added to the lower chamber of the trans-well chamber. The plates were incubated at 37 °C for 24 h. After the removement of all nonmigrated cells, the migrated cells were stained using crystal violet. A microscope was employed to capture the images and count the migrated cells.


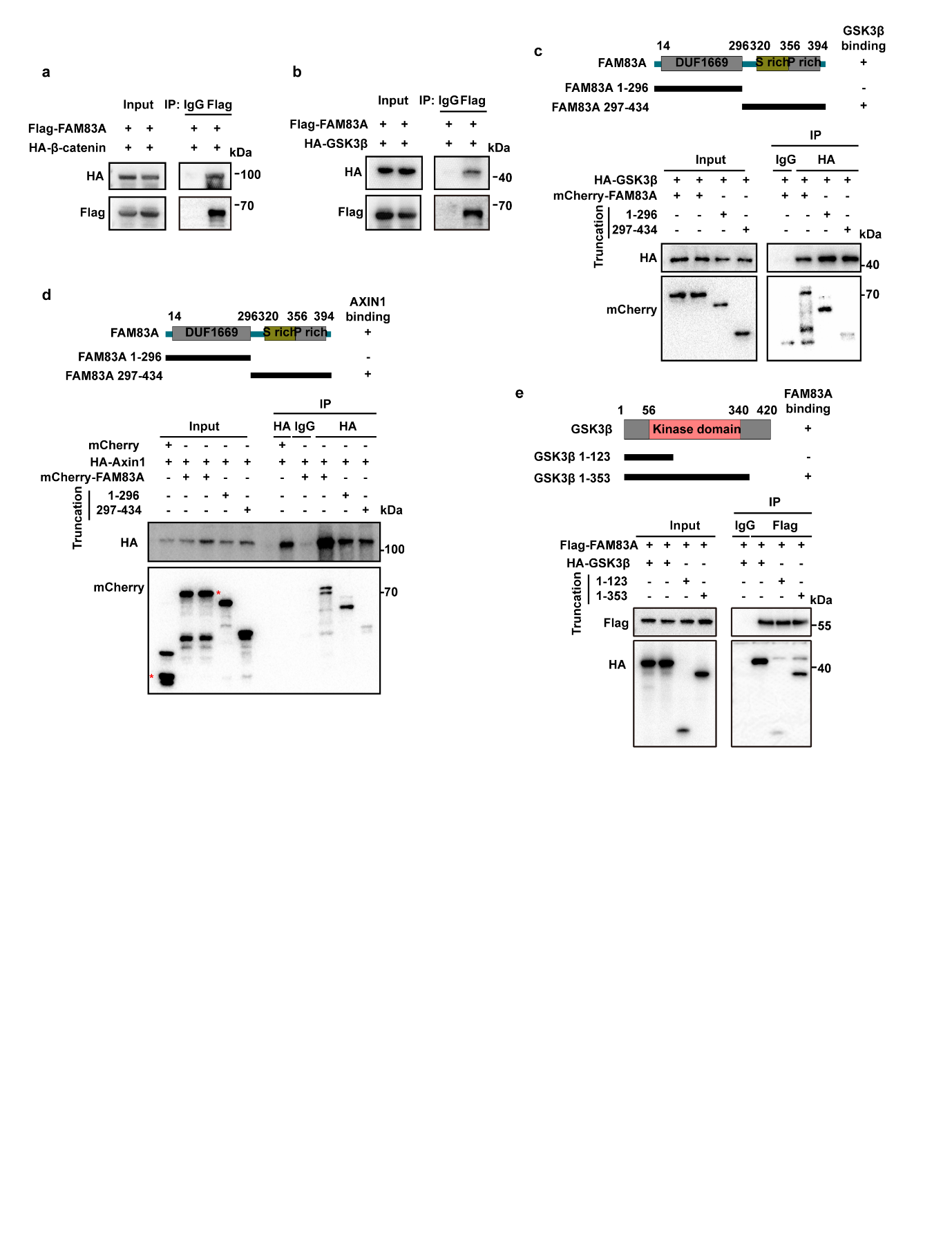


**Figure. S1 Interaction between FAM83A and β-catenin destruction complex, related to Fig.1.**

**a, b** The interaction between exogenous Flag-tagged FAM83A and HA-tagged β-catenin, GSK3β in HEK293T cells. **c** The schematic diagram of FAM83A truncation generation and FAM83A-GSK3β interacting domain. **d** The schematic diagram of FAM83A truncation generation and FAM83A-AXIN1 interacting domain. **e** The schematic diagram of GSK3β truncation generation and FAM83A-GSK3β interacting domain.


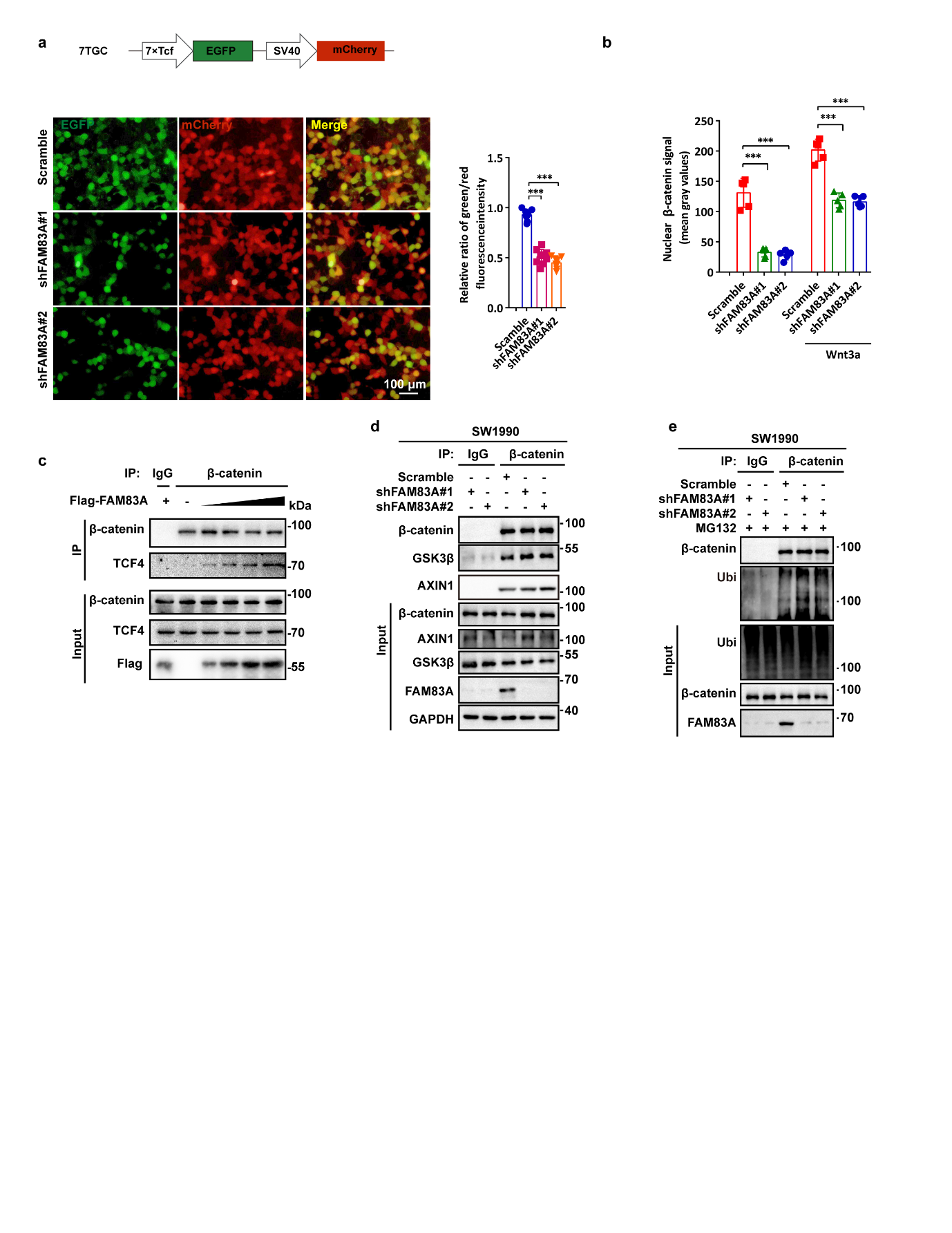


**Figure. S2 FAM83A promotes β-catenin-TCF4 interaction, related to Fig. 2 and Fig. 3.**

**a** The schematic diagram of 7TGC plasmid construction and the fluorescence images of PANC-1 cells with or without FAM83A knockdown. Relative ratio of green/red fluorescence intensity were quantified (n = 9). **b** The quantification of the nuclear β-catenin with or without FAM83A knockdown and Wnt3a treatment (100 ng/mL for 4 hours) in PANC-1 cells (n = 5). **c** FAM83A overexpression dose dependently increased the interaction between endogenous TCF4 and β-catenin in HEK293T cell lysates (n = 3). **d** FAM83A knockdown enhanced the interaction between endogenous GSK3β-β-catenin and AXIN1-β-catenin interaction in SW1990 cell lysates. Protein interactions were analyzed by western blotting (n = 3). **e** FAM83A knockdown promoted the level of β-catenin ubiquitination upon MG132 (10 μM) treatment in SW1990 cell lysates. Representative western blotting images were shown (n = 3). Protein interactions were analyzed by western blotting. *P < 0.05; **P < 0.01; ***P < 0.001. Data were presented as mean ± SD.


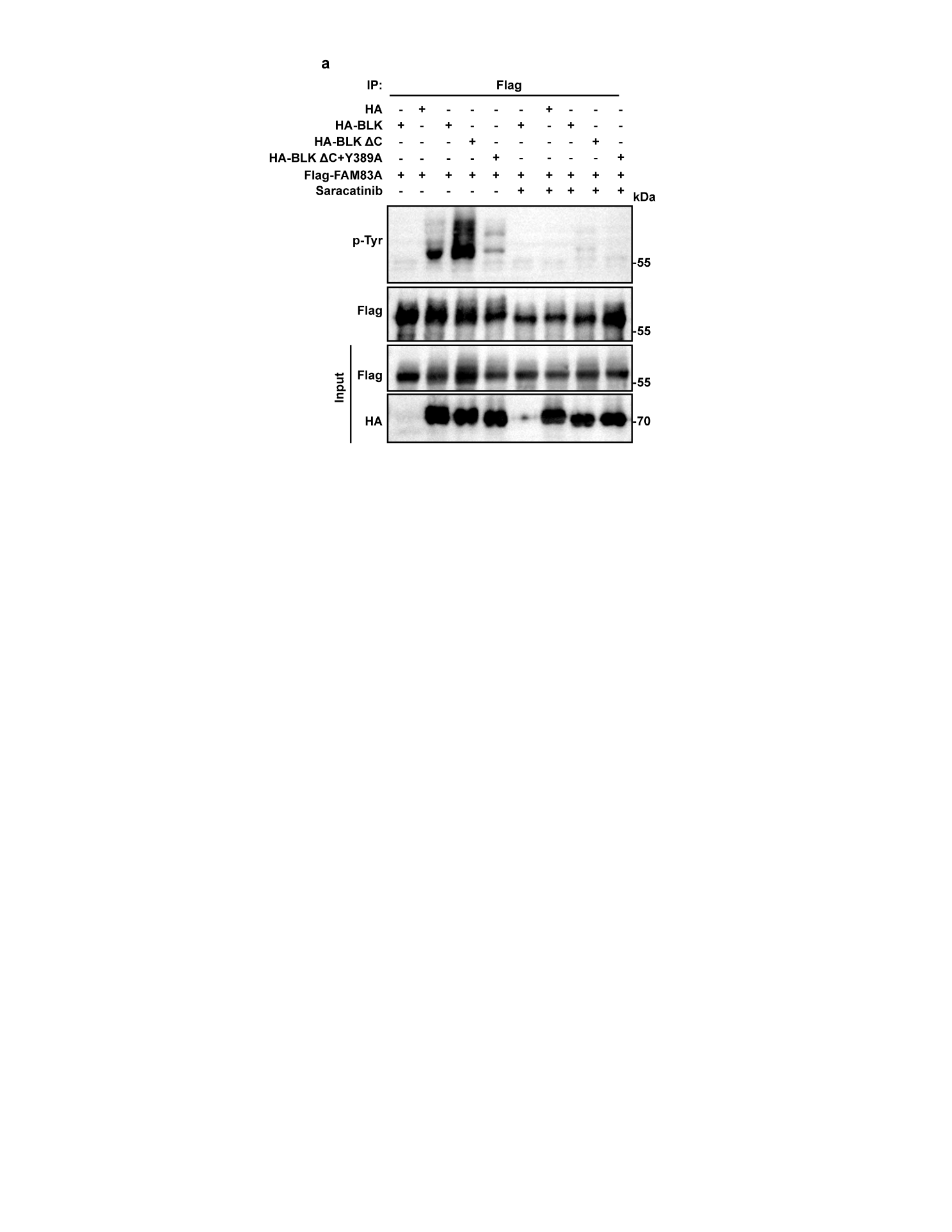


**Figure. S3 BLK phosphorylated FAM83A at Y138, related to Fig. 4.**

Level of total tyrosine phosphorylation of Flag-tagged FAM83A and its Y138A mutant in HEK293T cells transfected with either HA-tagged BLK wild-type, BLK kinase-dead mutant (Y389A) or BLK constitutive activated mutant with the deletion of the C-terminal auto-inhibitory domain (BLK ΔC) with or without BLK inhibitor saracatinib (10 μM) treatment (n = 3). Cell lysates were used for IP and western blotting with the indicated antibodies.


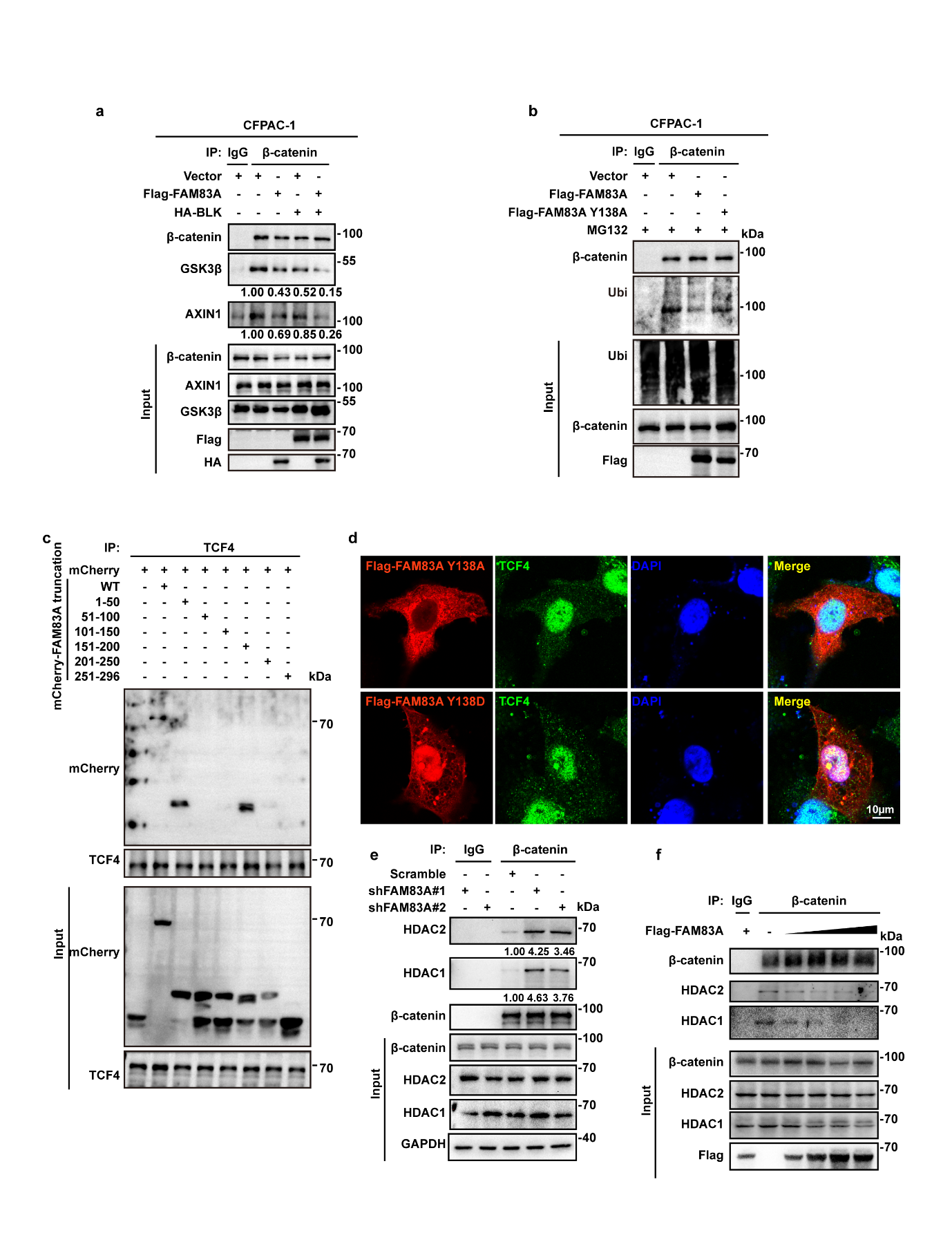


**Figure. S4 FAM83A binds TCF4 and inhibits TCF4-HDAC1/2 interaction, related to Fig. 5.**

**a** The interaction between endogenous β-catenin and GSK3β, AXIN1 after BLK kinase overexpression in CFPAC-1 cells. Protein interactions were analyzed by western blotting (n = 3). **b** The level of β-catenin ubiquitination with or without Flag-tagged FAM83A or Y138A mutants overexpression after MG132 treatment in CFPAC-1 cells. Cell lysates were used for IP and western blotting with the indicated antibodies (n = 3). **c** The interaction between the mCherry-tagged FAM83A truncation mutations with TCF4 in HEK293T cells. Cell lysates were used for IP and western blotting with the indicated antibodies. **d** The co-localization of Flag-tagged FAM83A Y138A, Y138D mutants with TCF4 were analyzed with confocal microscopy. **e, f** The interaction between HDAC1, HDAC2 and β-catenin after FAM83A knockdown in AsPC-1 cells or Flag-tagged FAM83A overexpression in HEK293T cells (n = 3). Cell lysates were used for IP and western blotting with the indicated antibodies.


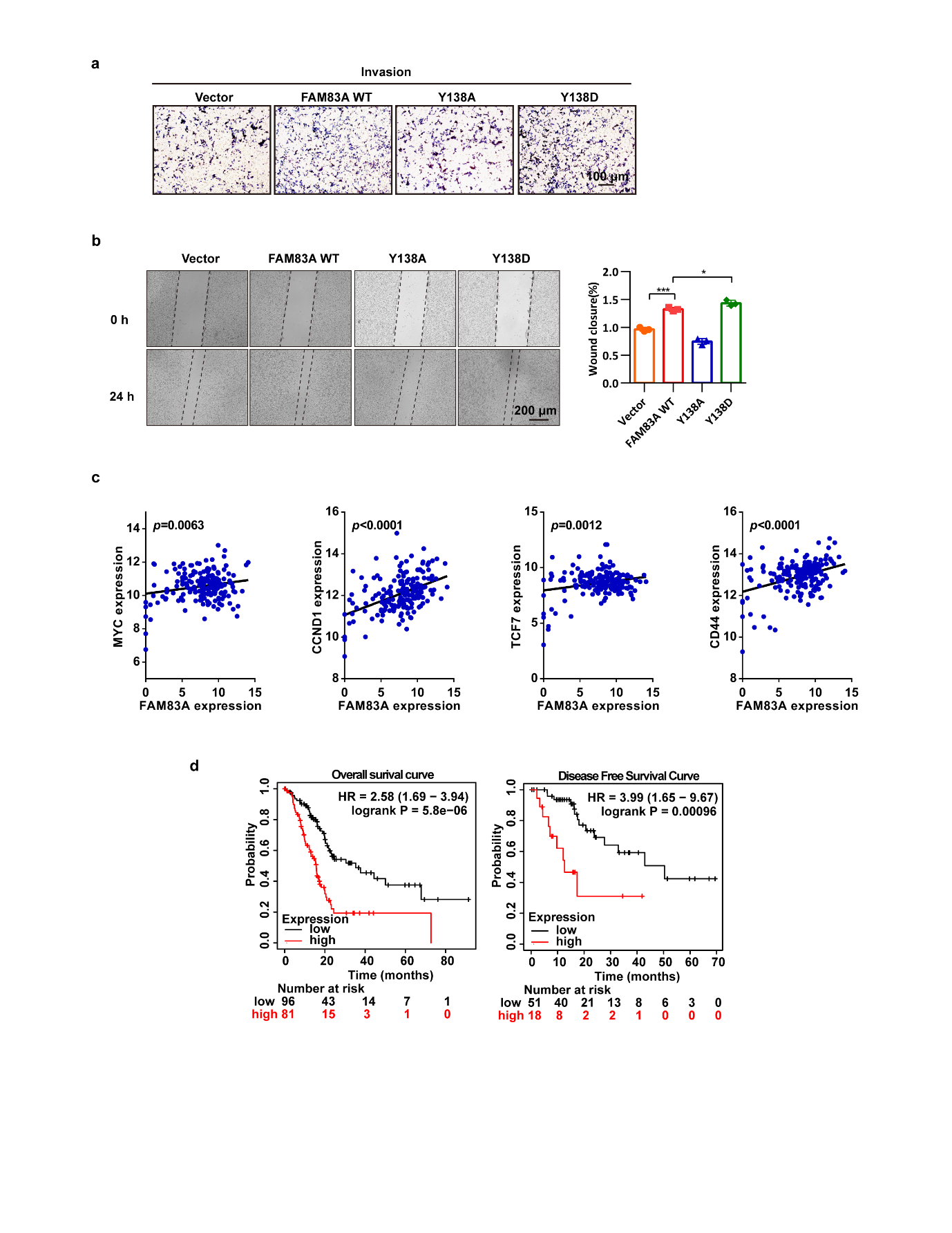


**Figure. S5 FAM83A Y138 phosphorylation promotes pancreatic cancer cell migration and invasion, related to Fig. 6 and Fig. 7.**

**a** Representative images of the trans-well assays with matrigel of PANC-1 cells stably expressed Flag-tagged wild-type FAM83A, Y138A or Y138D mutants (n = 3). **b** Representative images and quantification of the wound healing assays of PANC-1 cells stably expressed Flag-tagged wild-type FAM83A, Y138A or Y138D mutants (n = 3). **c** The correlation of the expression between FAM83A and Wnt/β-catenin target genes (n = 183). **d** The overall survival (OS) and disease-free survival (DFS) of pancreatic cancer patients with high or low FAM83A expression.


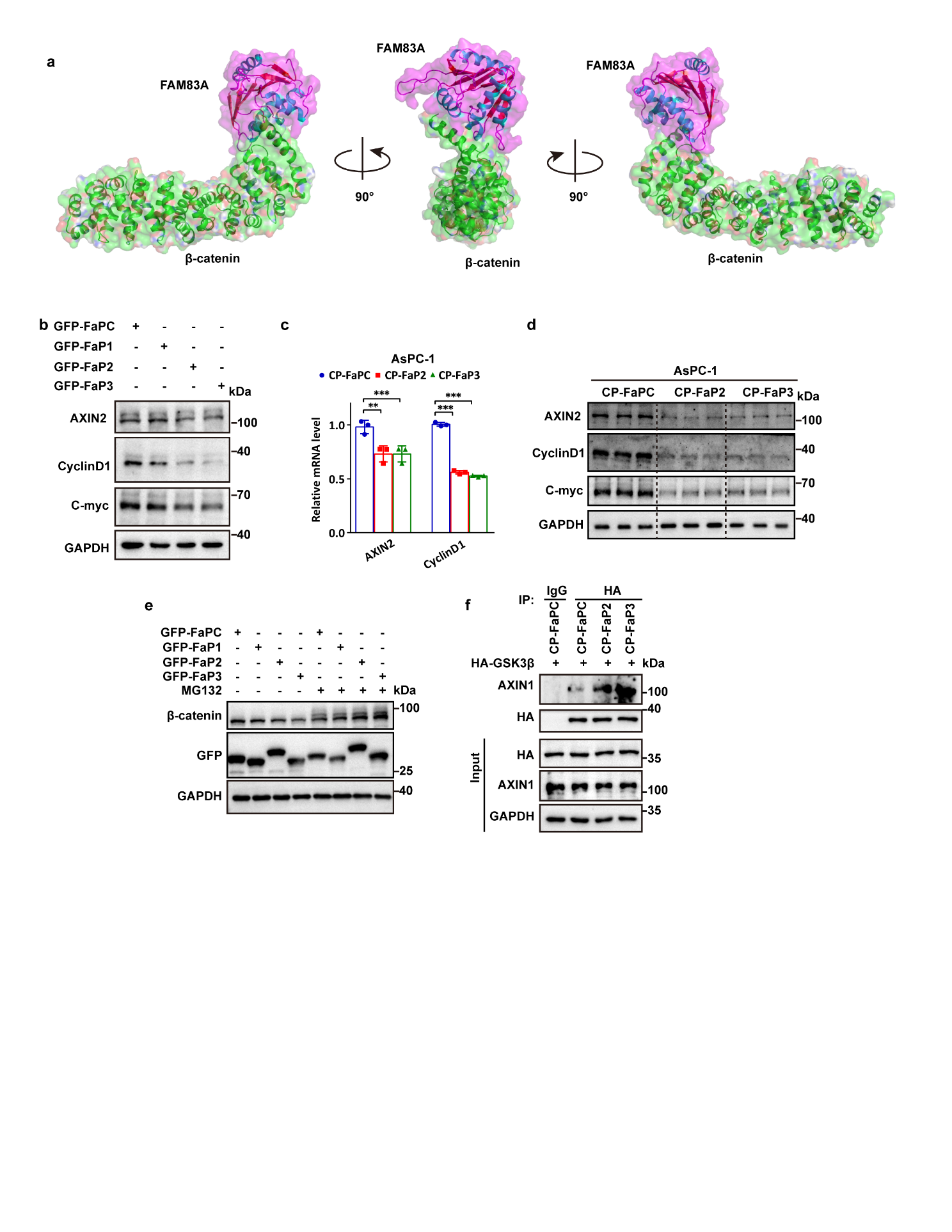


**Figure. S6 Peptides disrupting FAM83A-β-catenin interaction promote the assembly of β-catenin destruction complex, related to Fig. 8.**

**a** The predicted docking model of FAM83A (pdb:4urj) with β-catenin (pdb:1jpw). **b-d** Protein and mRNA level of Wnt target genes AXIN2, C-myc and CyclinD1 in AsPC-1 cells after control, CP-FaP2 and CP-FaP3 treatment or GFP-tagged FaP1, FaP2 and FaP3 transfection. Cell lysates were used for IP and western blotting with the indicated antibodies (n = 3). **e** The stability of β-catenin protein upon MG132 treatment in HEK293T cells after control, CP-FaP2 and CP-FaP3 treatment or GFP-tagged FaP1, FaP2 and FaP3 transfection (n = 3). Cell lysates were used for IP and western blotting with the indicated antibodies. **f** The interaction between AXIN1 and HA-tagged GSK3β in AsPC-1 cells after control, CP-FaP2 and CP-FaP3 treatment (n = 3). Cell lysates were used for IP and western blotting with the indicated antibodies.


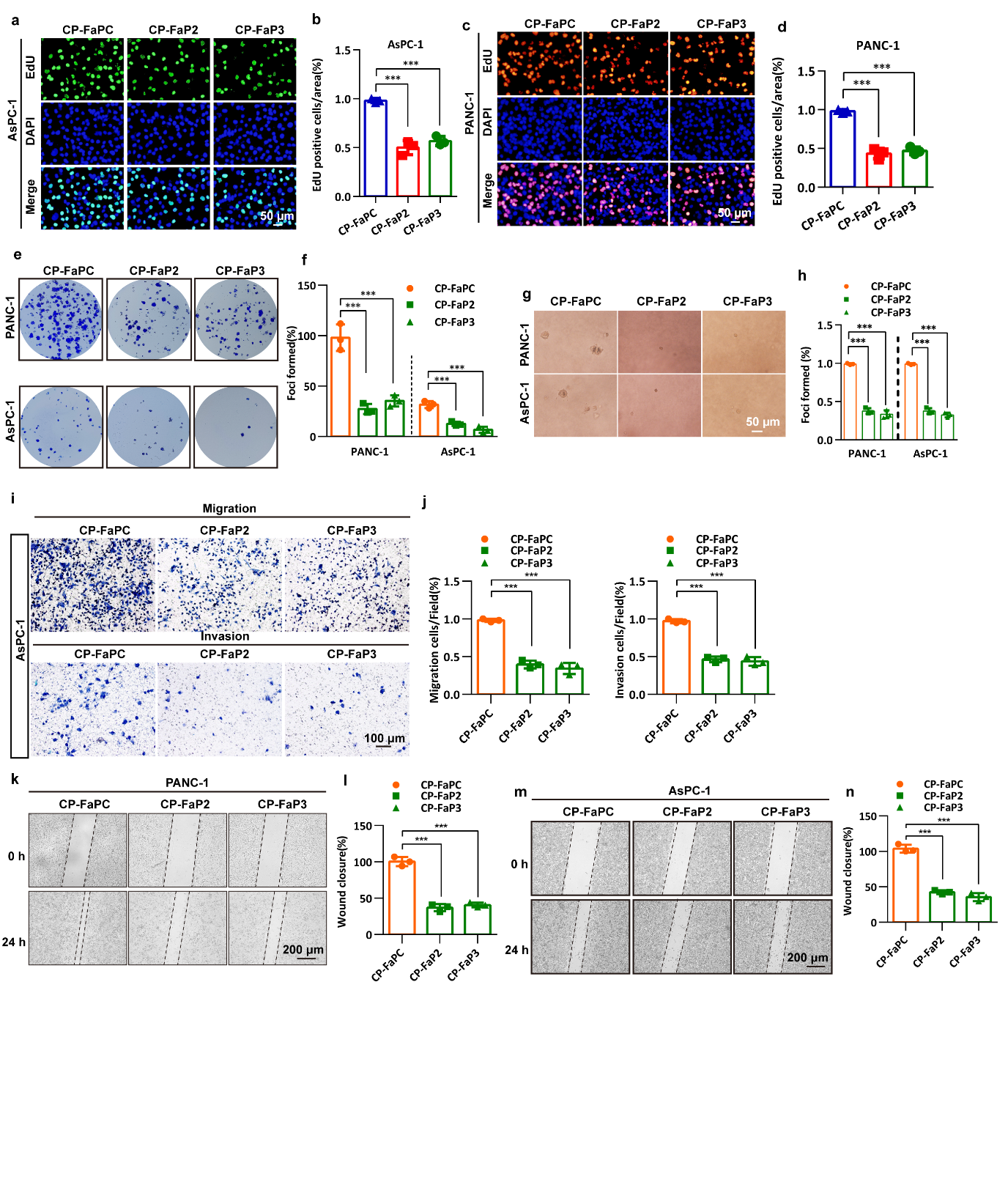


**Figure. S7 FAM83A-β-catenin interaction inhibitory peptides restrain pancreatic cancer cell proliferation and metastasis, related to Fig. 9.**

**a-d** Representative images and quantification of DNA synthesis in the PANC-1 and AsPC-1 cells treated with control, CP-FaP2 and CP-FaP3 peptides (n = 3). **e-h** Representative images and quantification of the soft agarose and plate colony formation assays of PANC-1 cells treated with control, CP-FaP2 and CP-FaP3 peptides (n = 3). **i, j** Representative images and quantification of the trans-well assays with or without matrigel of AsPC-1 cells treated with control, CP-FaP2 and CP-FaP3 peptides (n = 3). **k-n** Representative images and quantification of the wound healing assays of PANC-1 and AsPC-1 cells treated with control, CP-FaP2 and CP-FaP3 peptides (n = 3).

# References

1. Zhou C*, et al*. Identification of glycerol-3-phosphate dehydrogenase 1 as a tumour suppressor in human breast cancer. *Oncotarget* **8**, 101309-101324 (2017).

2. Livak KJ, Schmittgen TD. Analysis of relative gene expression data using real-time quantitative PCR and the 2(-Delta Delta C(T)) Method. *Methods* **25**, 402-408 (2001).

3. Zhou C*, et al*. Pygo2 functions as a prognostic factor for glioma due to its up-regulation of H3K4me3 and promotion of MLL1/MLL2 complex recruitment. *Sci Rep* **6**, 22066 (2016).

4. Villiers MB*, et al*. Peptide-protein microarrays and surface plasmon resonance detection: biosensors for versatile biomolecular interaction analysis. *Biosens Bioelectron* **26**, 1554-1559 (2010).

5. Zhou C*, et al*. Integral membrane protein 2A inhibits cell growth in human breast cancer via enhancing autophagy induction. *Cell Commun Signal* **17**, 105 (2019).

# 
